# Structure and dynamics of an α-fucosidase reveal a mechanism for highly efficient IgG transfucosylation

**DOI:** 10.1038/s41467-020-20044-z

**Published:** 2020-12-04

**Authors:** Erik H. Klontz, Chao Li, Kyle Kihn, James K. Fields, Dorothy Beckett, Greg A. Snyder, Patrick L. Wintrode, Daniel Deredge, Lai-Xi Wang, Eric J. Sundberg

**Affiliations:** 1 Institute of Human Virology, University of Maryland School of Medicine, Baltimore, MD 21201 USA; 2 Department of Microbiology & Immunology, University of Maryland School of Medicine, Baltimore, MD 21201 USA; 3 Program in Molecular Microbiology & Immunology, University of Maryland School of Medicine, Baltimore, MD 21201 USA; 4 Department of Chemistry and Biochemistry, University of Maryland, College Park, MD 20742 USA; 5 Department of Pharmaceutical Sciences, University of Maryland School of Pharmacy, College Park, MD 21201 USA; 6 Department of Medicine, University of Maryland School of Medicine, Baltimore, MD 21201 USA; 7 Present Address: Department of Biochemistry, Emory University School of Medicine, Atlanta, GA 30322 USA

**Keywords:** Enzyme mechanisms, X-ray crystallography, Hydrolases, Glycosides

## Abstract

Fucosylation is important for the function of many proteins with biotechnical and medical applications. Alpha-fucosidases comprise a large enzyme family that recognizes fucosylated substrates with diverse α-linkages on these proteins. *Lactobacillus casei* produces an α-fucosidase, called AlfC, with specificity towards α(1,6)-fucose, the only linkage found in human *N-*glycan core fucosylation. AlfC and certain point mutants thereof have been used to add and remove fucose from monoclonal antibody *N-*glycans, with significant impacts on their effector functions. Despite the potential uses for AlfC, little is known about its mechanism. Here, we present crystal structures of AlfC, combined with mutational and kinetic analyses, hydrogen–deuterium exchange mass spectrometry, molecular dynamic simulations, and transfucosylation experiments to define the molecular mechanisms of the activities of AlfC and its transfucosidase mutants. Our results indicate that AlfC creates an aromatic subsite adjacent to the active site that specifically accommodates GlcNAc in α(1,6)-linkages, suggest that enzymatic activity is controlled by distinct open and closed conformations of an active-site loop, with certain mutations shifting the equilibrium towards open conformations to promote transfucosylation over hydrolysis, and provide a potentially generalizable framework for the rational creation of AlfC transfucosidase mutants.

## Abstract (web summary)

AlfC transfucosidase is used to modulate fucosylation of glycans decorating monoclonal antibodies. Herein, structural and biophysical characterization reveals the enzymatic mechanism of AlfC and a blueprint for the design of AlfC mutants with novel specificities and functions.

## Introduction

Fucosylation is a common post-translational modification that has important roles in human physiology and pathology, including ABO blood grouping, antibody effector functions, cancer progression, and lymphocyte development and adhesion^1,2^. As such, fucose-modifying enzymes are relevant to many aspects of human health. Alpha-fucosidases are hydrolytic enzymes active on α-l-fucosyl linkages, classified as members of the glycoside hydrolase families 29 and 95. GH95 enzymes are a small family of inverting α-fucosidases with activity on α(1,2)-fucosyl galactose^3^. In contrast, GH29 enzymes are a broad family of retaining fucosidases with activity on α(1,2)-, α(1,3)-, α(1,4)- and α(1,6)-fucosyl linkages. GH29 has been further divided into subfamilies A and B, with GH29A active on a wide range of linkages, while GH29B is specific for branched α(1,3)- and α(1,4)-fucosyl linkages^4–6^. Although the molecular basis for substrate recognition and catalysis has been described in some GH29B enzymes^7^, it is still poorly understood in GH29A enzymes. This is in part due to the diversity in substrate specificity of GH29A enzymes, where enzymes with different linkage specificities necessarily have different mechanisms for binding their substrates. It is further complicated by the fact that a key catalytic residue, specifically the general acid/base, cannot be predicted based on sequence alignments^5^. Although the catalytic mechanism has been described in one GH29A enzyme (BT2970)^8^, it is unclear if the same mechanism occurs in other GH29A enzymes. Although it would be unusual for enzymes within the same family to have different mechanisms, chemical tests including azide rescue have implicated residues in three different positions as the general acid/base in various GH29A enzymes^6,8–10^ (Supplementary Table S1). Therefore, detailed structural and kinetic analyses of GH29A enzymes is paramount to understanding how these enzymes recognize and hydrolyze their substrates.

*Lactobacillus casei* produces a GH29A α-fucosidase, AlfC, that is ~10^4^-fold more active on α(1-6)-fucosyl linkages compared to other linkages^11,12^. This enzyme is particularly relevant because α(1-6) linkages form the basis of human core fucosylation, which significantly impacts the function of glycoproteins such as antibodies^13^. As such, AlfC and designer point mutants have been created previously to specifically alter the core fucosylation of glycoproteins^12^. The mutation of a glutamic acid residue at position 274 to alanine (AlfC_E274A_) has been shown to convert AlfC from a hydrolase into an efficient transfucosidase that can transfer a fucose moiety to an acceptor (e.g., *N*-acetylglucosamine [GlcNAc]) from a simple fucosyl donor (e.g., α-fucosyl fluoride) with nearly quantitative yields^12^. Because this residue aligns by sequence with the general acid/base of the human α-fucosidase FucA1, it was presumed that this residue was the acid/base in AlfC, with AlfC_E274A_ functioning as a fucoligase^12^. However, this corresponds by sequence alignment to a different residue than the known acid/base in the closely related and well-studied GH29A enzyme BT2970^8^ (Supplementary Table S1). No crystal structure has ever implicated a GH29A enzyme to have an acid/base residue other than the one corresponding to that of BT2970. Therefore, several uncertainties remain, including whether or not GH29A enzymes have diverse mechanisms, what determines the linkage specificity of AlfC and other GH29A enzymes, and why AlfC_E274A_ is an efficient transfucosidase. In the current study, we employed X-ray crystallography, mutational and kinetic analyses, hydrogen–deuterium exchange mass spectrometry, molecular dynamic simulations, and transfucosylation experiments to define the molecular mechanisms of the activities of AlfC and its transfucosidase mutants. Our results suggest that AlfC employs a conserved hydrolytic mechanism with a specific subsite that accommodates GlcNAc in α(1,6)-linkages, while AlfC tranfucosidase mutants work by shifting equilibrium between open and closed conformations of an active-site loop.

## Results

### Defucosylation of IgG

**Fig. 1:**
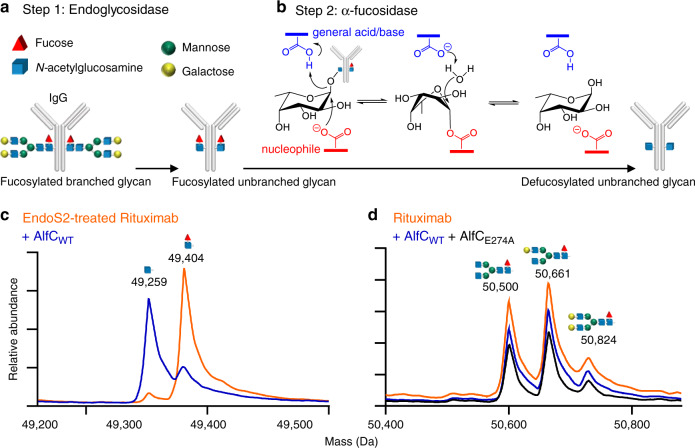
Defucosylation of IgG. **a** Antibodies are treated with an endoglycosidase, leaving an *N-*linked disaccharide. **b** Alpha-fucosidase defucosylation proceeds through a Koshland double-displacement reaction with retaining stereochemistry. **c** Mass spectrometry of AlfC-treated Rituximab in the presence of EndoS2; **d** AlfC-treated Rituximab in the absence of EndoS2. Mass spectrometry plots have been re-traced and overlaid for clarity. Source data are provided as a Source Data file.

Not all α-fucosidases have hydrolytic activity on the core fucose of antibodies, and efficient hydrolysis has only been reported in one other case^14^. However, in both cases, antibodies were first treated with an endoglycosidase, such as EndoS or EndoS2, to remove all of the carbohydrate residues following the *N*-linked GlcNAc and its accompanying core fucose, trimming the *N-*glycan to a Fuc-α(1,6)-GlcNAc disaccharide (Fig. 1a)^12,14–16^. Next, α-fucosidases have been used to hydrolyze the core fucose through a Koshland double-displacement reaction with retaining stereochemistry (Fig. 1b). No α-fucosidase has been shown to defucosylate intact branched glycosylated IgG. The reported ability of the transfucosidase mutant AlfC_E274A_ to catalyze the addition of fucose to afucosylated branched glycosylated IgG^12^ led us to wonder if wild-type AlfC could defucosylate intact branched glycosylated IgG. We incubated AlfC_WT_ with Rituximab, a clinically approved IgG1 antibody bearing a fucosylated complex biantennary glycan, and monitored reaction progression using mass spectrometry. Consistent with previous reports, AlfC efficiently removed fucose from Rituximab after overnight incubation when an endoglycosidase (EndoS2) is present^14^ (Fig. 1c). In stark contrast, neither AlfC_WT_ nor AlfC_E274A_ had any detectable activity in the absence of the endoglycosidase as much as 1 month later (Fig. 1d), despite the fact that mutant E274A could add fucose to the core GlcNAc moiety of an intact and fully glycosylated antibody^12^. The seeming paradox that a point mutant (E274A) could create a product that neither it nor the wild-type enzyme can hydrolyze led us to explore the molecular basis for enzymatic activity by AlfC_WT_ and point mutants.

### Overall structure of AlfC

**Fig. 2:**
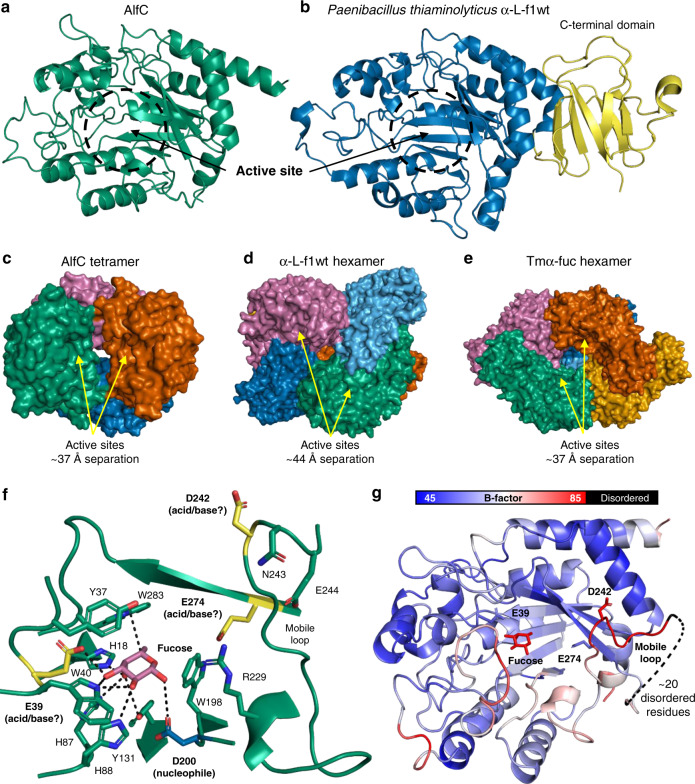
Structure of AlfC and similar α-fucosidases. **a** Structure of AlfC with active-site annotated. **b** Structure of closest homolog α-L-f1wt, exhibiting a C-terminal domain present in most α-fucosidases, but absent in AlfC. **c** Tetrameric assembly of AlfC compared to **d** α-l-f1wt (hexamer) and **e** Tmα-fuc (hexamer). Active-site separation is measured by the distance between catalytic nucleophiles. **f** Structure of AlfC active site with l-fucose bound. Enzyme contacts are shown by dashed lines. The catalytic nucleophile is shown in blue, while lead candidates for the general acid/base are shown in yellow. A mobile loop containing D242 is annotated. **g** Cartoon representation of AlfC structure colored by B-factor, highlighting high B-factors in the mobile loop that precedes a disordered region.

To gain insight into the structure-function relationship of AlfC, we solved the 2.5 Å resolution X-ray crystal structure of full-length wild-type AlfC (Supplementary Table S2). AlfC is a single-domain enzyme that adopts a (β/α)_8_ fold typical of GH29 enzymes, with an active site at the center of the barrel (Fig. 2a). According to the DALI server^17^, AlfC is most similar structurally to α-l-f1wt produced by the bacteria *Paenibacillus thiaminolyticus* (PDB 6GN6; *Z*-score = 38.5, C_α_RMSD = 2.2 Å, amino acid identity = 35%)^18^. However, unlike this enzyme and most other α-fucosidases, AlfC is missing the C-terminal domain often hypothesized to be a carbohydrate-binding module (Fig. 2b). AlfC crystallized as a tetramer, with opposing active sites separated by ~37 Å (Fig. 2c). The tetrameric nature of AlfC is consistent with previously published gel filtration experiments^11^ and the multimeric nature of other α-fucosidases, such as the closely related α-L-f1wt (hexamer; Fig. 2d) and well-studied TmαFuc (hexamer; Fig. 2e)^19–22^. To confirm this, we performed sedimentation equilibrium analysis, which also indicates that AlfC exists as a tetramer in solution (Supplementary Fig. S1). The significance of the multimeric nature of α-fucosidases is unclear, and in only one case has the quaternary assembly been reported to have functional significance, with the active site of one monomer complemented by a segment from an adjacent monomer^18^. Although active-site complementation is not seen in AlfC, it remains unknown whether or not cross-talk can occur between active sites.

### Structure and dynamics of the AlfC active site

To better understand the catalytic mechanism of AlfC_WT_ and its point mutants, we solved the X-ray crystal structure of AlfC_WT_ into which we soaked l-fucose (Table S2). In these crystals, fucose produced no substantial conformational changes, with a C_α_RMSD = 0.14 Å between the fucose-bound and -unbound structures. Fucose binds in an orientation identical to other previously determined fucosidase crystal structures^20,23,24^. Specifically, H18, H87, and Y131 coordinate O4 of fucose; E39 and W40 coordinate O3; W40 and H88 coordinate O2; and nucleophile D200 interacts with O1. W283 and W198 contribute to a hydrophobic pocket that accommodates C6 (Fig. 2f).

The mechanism of the closely related BT2970 has been characterized previously^8^. Hydrolysis proceeds through a classical Koshland double-displacement reaction, with a ^1^C_4_ ↔ ^3^H_4_ ↔ ^3^S_1_ conformational itinerary^8,20^ (Fig. 1b). In this mechanism, the catalytic nucleophile and general acid/base oxygens (both typically contributed by aspartate and glutamate residues) are separated by ~5.5 Å (this results in a C_α_–C_α_ distance of ~12 Å, as described later). In AlfC, the nucleophile (D200) is conserved in both sequence and structure. However, no general acid/base residue is present within the expected ~5.5 Å distance. Structure and sequence homology implicate three main candidates for the general acid/base residue: E39, E274, and D242 (Fig. 2f).

The sequence equivalent of E39 has been implicated as the acid/base through chemical experiments in the α-fucosidases from *Sulfolobus sulfataricus* (Ssα-fuc)^9^ and *Nephilingis cruentata* (NcFuc) (Supplementary Table S1)^6^. However, there is no structural evidence to support this assignment in any fucosidase, and O_δ_ of this residue in AlfC is ~9 Å from the nucleophile O_γ_, with no plausible access to the reactive C1 of fucose. Its location on an α-helix with very low B-factors (Fig. 2g) suggests it is unlikely to unfold to move closer to C1 of fucose, while its hydrogen bond with O3 makes it far more likely to be involved in fucose binding rather than catalysis.

The sequence equivalent of E274 has been implicated as the general acid/base by chemical evidence in the α-fucosidases from *Homo sapiens* (FucA1)^10^ and *Elizabethkingia meningoseptica* (cFase I) (Supplementary Table S1)^25^. However, as for E39, no structural evidence exists to support this assignment in any fucosidase. In AlfC, O_δ_ of this residue is ~12 Å from the nucleophile O_γ_, with W198 separating the two residues. Furthermore, its location on a core β-strand with very low B-factors (Fig. 2g) makes it unlikely that a conformational change could place it within ~5.5 Å of the nucleophile to support a direct role in catalysis.

**Fig. 3:**
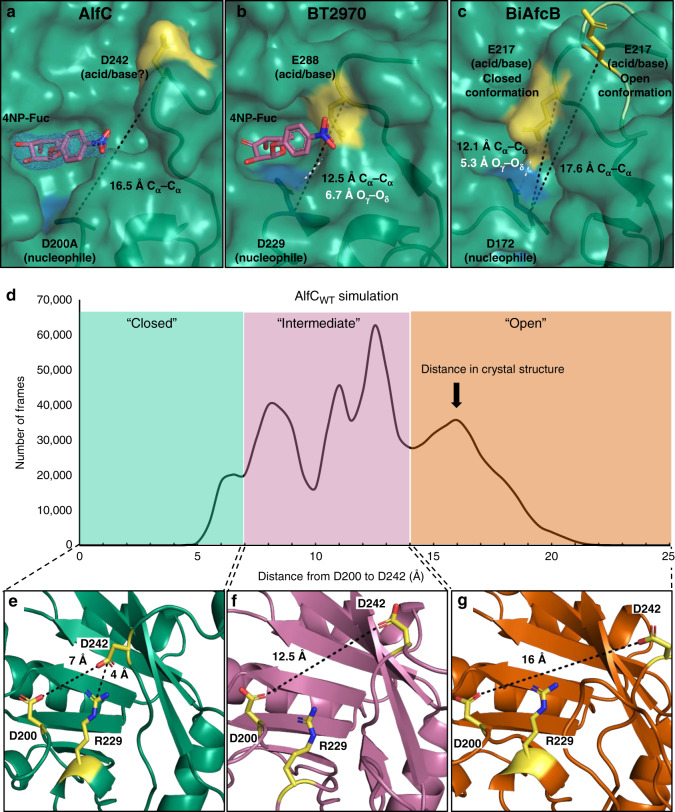
Structure and dynamics of AlfC D242 loop. **a** Structure of AlfC_D200A_ bound to 4-nitrophenyl-α-l-fucopyranoside (4NP-fuc) in an open conformation. The blue mesh represents a composite omit map of electron density surrounding 4NP-fuc, contoured to 1.5 *σ*, and carved to 1.7 Å. **b** Structure of BT2970 (PDB 2WVU) with the same substrate in a closed conformation. **c** Structure of BiAfcB exhibiting open (PDB 3MO4) and closed (PDB 3UES) conformations. **d** Molecular dynamic simulation of AlfC_WT_ with frames plotted by the distance separating AlfC D200 and D242 O_δ_. **e**, **f** Representative frames depicting AlfC conformations. **e** Closed conformation depicting <7.5 Å distance between D200 and D242, with a salt bridge between R229 and D242; **f** intermediate conformation; **g** open conformation. Source data are provided as a Source Data file.

The structural equivalent of D242 has been implicated as the general acid/base by structural and/or chemical evidence in nine α-fucosidases to-date (BT2192, BT3798, BiAfcB^7^, BACOVA_04357, GH29_0940^26^, FgFCO1^23^, α-L-f1wt^18^, αTm-Fuc^20^, and BT2970^8^) making it the presumed general acid/base residue if AlfC shares a similar mechanism. The D242 O_γ_ atom is ~19 Å away but located on a loop with very high B-factors that extends into a disordered region in our crystal structures, which together suggest a high degree of flexibility (Fig. 2g). It is common for α-fucosidases to be crystallized in either an open conformation, as seen in these structures of AlfC, where the presumed general acid/base is far from the active site, or in a closed conformation, where it moves into the active site to support catalysis^7^. To see if the presence of a substrate would induce a conformational change, we solved the X-ray crystal structure of catalytically inactive AlfC_D200A_ in complex with the chromogenic substrate 4-nitrophenyl-α-l-fucopyranoside (4NP-fuc), which is frequently used in kinetic studies of α-fucosidases, and was used in the combined structural/quantum mechanical analysis of BT2970 to define the mechanism of this enzyme^8^ (Supplementary Table S1). We soaked this substrate into apo AlfC_D200A_ crystals and observed clear electron density for it. However, despite the bound substrate, we observed no conformational changes in any of the acid/base candidates, and the C_α_ of D200A and D242 were still ~16.5 Å apart (Fig. 3a). 4NP-fuc bound in an orientation nearly identical to that seen in BT2970, where the C_α_ of the nucleophile and acid/base are separated by ~12.5 Å, in a conformation compatible with catalysis (Fig. 3b)^8^. In BiAfcB, it has been observed that the C_α_ of the acid/base can move from ~17.6 Å away from the nucleophile C_α_, to only ~12.1 Å away (Fig. 3c)^7^. The same conformational change in AlfC would place the C_α_ of D242 only ~11 Å from the nucleophile C_α_, in a position compatible with catalysis. Therefore, structural homology suggests the existence of open and closed states of AlfC.

In order to understand if D242 or one of the other acid/base candidates is capable of moving into the active site to support catalysis, we probed the dynamics of AlfC using molecular dynamic simulations. As expected, these simulations suggest that AlfC adopts several distinct conformations of D242 and the loop on which it is situated (Fig. 3d). In ~11% of the 840,000 pairwise distances (100 ns simulation with 52,500 frames, 4 monomers and 4 pairwise O_δ_ distances per monomer), AlfC adopts a closed conformation, where a D242 O_δ_ is less than 7.5 Å from the nucleophile D200 O_δ_, in a position compatible with catalysis. In this conformation, R229 forms a salt bridge with D242, in a position in which it could help recruit D242 to the active site (Fig. 3e). This may be a key interaction of the closed conformation, as probability graphs based on pairwise distance distributions suggest that it is nearly ubiquitously present in the closed conformation (Supplementary Fig. S2). Although empirical force fields tend to overestimate the strength of salt-bridges, this interaction is nearly identical to the salt bridge observed in the crystal structure of BT2970, where R262 recruits the acid/base E288 to the active site (Supplementary Fig. S2). In ~57% of frames, AlfC adopts intermediate conformations, where D242 is separated from D200 by 8–14 Å, and not interacting with R229 (Fig. 3f). Finally, in ~31% of frames, AlfC adopts open conformations, where D242 is separated from D200 by more than 14 Å, resembling the conformation observed in the abovementioned crystal structures (Figs. 2a and 3g). In contrast to the catalytically active conformations observed relative to D242, the other acid/base candidate residues, E39 and E274, never sample active conformations, instead of drifting further from D200 in over 90% of frames and never interacting with R229 (Supplementary Fig. S3). Altogether, when combined with our crystallographic data, molecular dynamics suggest that the catalytically active state of AlfC is a closed conformation where D242 moves in to act as the general acid/base.

### Kinetic analysis of AlfC

Because we did not directly observe a residue acting as an acid/base in our crystal structures, we sought to determine it independently by chemical means. The most common method for this is azide rescue^27^. Briefly, when the general acid/base is mutated to a small, nonionizable residue such as alanine, an azide ion can occupy the cavity previously occupied by the acid/base side chain. If the acid/base mutant enzyme is supplied with a substrate with a strong leaving group, the first step of the reaction (glycosylation) can still occur without acid catalysis. The enzyme then gets trapped with a covalent fucosyl-enzyme intermediate that it cannot hydrolyze without base assistance. However, when azide is supplied, it can act as a strong nucleophile, attacking the enzyme intermediate, and regenerating the enzyme. Therefore, mutating the acid/base results in a large (~10^4^-fold) decrease in *k*_cat_ and a decrease in *K*_M_ (due to accumulation of intermediate) that is partially reversed by the addition of azide.

**Fig. 4:**
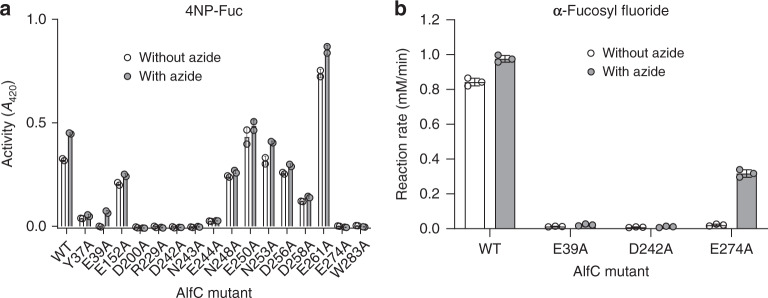
Activity and azide rescue of AlfC_WT_ and point mutants. **a** Reactions were measured in duplicate on separate samples with 4NP-Fuc as substrate. **b** Reactions were measured in triplicate on separate samples with α-fucosyl fluoride as substrate. Error bars represent the standard deviation of *n* = 3 independent experiments. Data are presented as mean values ± SEM. Source data are provided as a Source Data file.

We took an unbiased approach, individually mutating every aspartic acid and glutamic acid within ~15 Å of the active site. This included a large disordered region near the active site (residues 247–266; Fig. 2g). We also mutated every asparagine residue in the same region, as asparagine can be activated by an adjacent residue to function as a general acid/base in some GH95 α-fucosidases^28^. We additionally mutated several other residues near the active site (including tyrosine, tryptophan, and arginine residues) that might have important mechanistic roles. To screen for loss of activity consistent with a catalytic role, we performed a colorimetric endpoint assay using 4NP-fuc. Using this assay, activity was undetectable in seven mutants (Fig. 4a). These mutants included the nucleophile (D200), the active-site arginine (R229), all three main acid/base candidates (E39, D242, E274), an asparagine (N243) adjacent to D242, and an active-site tryptophan (W283) (Fig. 2f). The activity could be measurably rescued by sodium azide only in the case of E39A. The activity of one mutant (E261A) exceeded wild-type activity by ~2-fold.

To further characterize these mutants, we performed detailed kinetic analyses to establish kinetic parameters (*k*_cat_ and *K*_M_) (Table 1). AlfC_WT_ has low catalytic efficiency for 4NP-fuc, with a turnover rate (*k*_cat_) = 1.4 s^−1^ and a *K*_M_ = 0.7 mM, yielding a *k*_cat_/*K*_M_ around 2 s^−1^ mM^−1^. This is unsurprising, as α-fucosidases have been reported to have a wide range of catalytic efficiencies (*k*_cat_/*K*_M_) for this substrate, as high as ~10^4^ s^−1^ mM^−1^ in SSα-fuc^9^, and unmeasurably low in subfamily B enzymes. The mutation E261A is more active than wild-type due to a ~3-fold increase in turnover rate. The presence of sodium azide also produces a mild turnover rate enhancement of about 25% for the wild-type enzyme. In contrast, it produces a 10-fold turnover rate enhancement for E39A, the only mutant to exhibit such an increase. However, this mutation produced a ~18-fold increase in *K*_M_, rather than the expected decrease for a residue acting as the acid/base^27^. This observation, combined with the ~7-fold decrease in *K*_M_ in the presence of sodium azide, suggests that azide ions might be occupying the cavity formed by E39A to promote binding of 4NP-fuc, and subsequent conformational changes needed to promote catalysis. This is consistent with the structure demonstrating a hydrogen bond between this residue and O3 of fucose (Fig. 2f). There was also a large (10^3^-fold) decrease in turnover for N243A, implicating a role for this residue in catalysis. The nucleophile mutant D200A resulted in undetectable activity (>10^8^-fold decrease), as did R229A, suggesting that the salt bridge it forms with D242 in molecular dynamic simulations might be a critical interaction.

**Table 1:** Kinetic parameters of AlfC_WT_ and point mutants.

Enzyme	*k*_cat_ (s^−1^)	*K*_M_ (mM)	*k*_cat_/*K*_M_ (s^−1^ mM^−1^)
E261A	4.27 ± 0.09	0.77 ± 0.06	5.5
Wild-type + azide	1.77 ± 0.01	0.72 ± 0.02	2.4
Wild-type	1.38 ± 0.02	0.70 ± 0.03	2.0
E39A + azide	6.9 (±0.1) × 10^−1^	2.3 ± 0.1	3.0 ×10^−1^
E244A	1.23 (±0.02) × 10^−1^	0.58 ± 0.03	2.1 × 10^−1^
Y37A	5.1 (±0.1) × 10^−1^	3.8 ± 0.2	1.3 × 10^−1^
N243A	3.21 (±0.05) × 10^−3^	0.64 ± 0.04	5.0 × 10^−3^
E39A	6.74 (±0.05) × 10^−2^	14 ± 2	4.6 × 10^−3^
W283A	1.91 (±0.08) × 10^−2^	7.2 ± 0.6	2.6 × 10^−3^
E274A	2.33 (±0.04) × 10^−3^	1.14 ± 0.07	2.0 × 10^−3^
D242A	6.5 (±0.5) × 10^−4^	0.84 ± 0.2	7.7 × 10^−4^
R229A	ND	ND	ND
D200A	ND	ND	ND

Kinetic parameters measured using 4-nitrophenyl-α-L-fucopyranoside at room temperature in 100 mM sodium citrate pH 5.8.

*ND* not determined.

Because of the unexpected azide rescue results with 4NP-fuc, we repeated it for the most likely acid/base candidates (E39, D242, E274) using the substrate 2-deoxy-2-fluoro-α-l-fucosyl fluoride (α-fucosyl fluoride), which bears a much stronger fluoride leaving group (pKa HF = 3.2)^22^. The reaction, monitored by ^19^F NMR (Supplementary Fig. S4), revealed that only E274A could be rescued by the addition of azide, reaching a reaction speed of about one-third of the wild-type speed (Fig. 4b). To determine if an α-configured rescue product is formed by E274A azide rescue, the product was analyzed by ^1^H NMR (Supplementary Fig. S5) and ^13^C NMR (Supplementary Fig. S6), which revealed a small coupling constant (*J* = 4.0 Hz), consistent with the expected α-anomeric configuration (Supplementary Fig. S7). These results are difficult to reconcile with crystal structures that show E274 ~12 Å from the active site (Fig. 2f), located on a β-strand with very low B-factors (Fig. 2g), and unable to move closer during simulations (Supplementary Fig. S3). It is possible that, as with E39A, azide fills a cavity to promote conformational changes that make rescue possible.

### Alpha(1,6)-specificity of AlfC

**Fig. 5:**
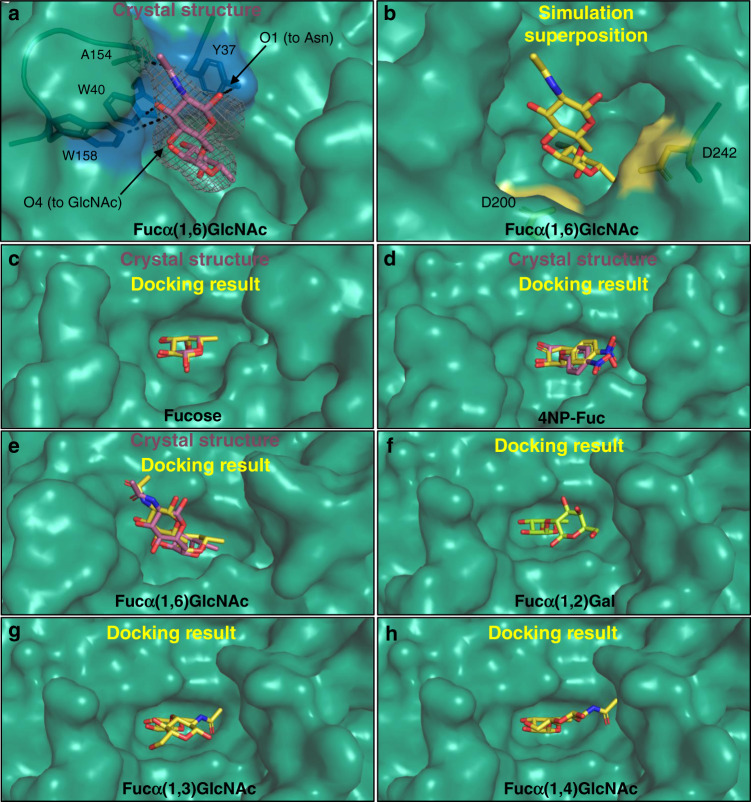
Structural basis of AlfC α(1,6)-specificity. **a** Crystal structure of AlfC_D200A_ with Fucα(1,6)GlcNAc. The gray mesh represents a composite omit map of electron density surrounding the ligand, contoured to 1.5 *σ*, and carved to 2.2 Å. An aromatic pocket that accommodates the GlcNAc +1 moiety is colored blue. **b** Superposition of Fucα(1,6)GlcNAc crystal structure over the closed state from molecular dynamic simulation (Fig. 3e) depicts a well-accommodated substrate. **c**–**e** Molecular docking of AlfC ligands (yellow) compared to crystal structures (magenta). Accuracy of docking shown by structural comparisons of **c** fucose; **d** 4NP-Fuc; **e** Fucα(1,6)GlcNAc. **f**–**h** Molecular docking results for inefficient substrates show +1 moiety outside the aromatic pocket, near the D242 loop. **f** Result for Fucα(1,2)Gal; **g** Fucα(1,3)GlcNAc; and **h** Fucα(1,4)GlcNAc.

AlfC is 10^3^–10^4^ fold more active on α(1,6)-linked disaccharides compared to α(1,2)-, α(1,3)-, and α(1,4)-linkages, and has no activity on polysaccharides greater than two residues^11^. In order to understand the α(1,6) specificity of AlfC, we solved the crystal structure of AlfC_D200A_ bound to its preferred substrate, Fucα(1,6)GlcNAc (Fig. 5a and Supplementary Table S2). With fucose in the active site, GlcNAc sits in an aromatic subsite adjacent to the active site, formed by residues Y37, W40, and W158. This binding mode furthermore allows oxygen of the *N*-acetyl group to hydrogen bond with the backbone nitrogen of A154. *N*-linkages of this disaccharide (as seen on EndoS-treated IgG) can be readily accommodated, as C1 of GlcNAc (which connects to Asn297 of IgG) points away from the surface of the enzyme. In contrast, AlfC cannot accommodate longer carbohydrate linkages (as seen in untreated IgG), because O4 of GlcNAc (which connects to another GlcNAc) would point directly toward the surface of the enzyme, creating steric clashes for any polysaccharide longer than two residues. When the Fucα(1,6)GlcNAc crystal structure is superimposed over the closed state conformation from the molecular dynamics simulation, it is apparent that the closed state still accommodates the glycan well, with D200 and D242 flanking the reactive C1 of fucose (Fig. 5b).

In order to understand why AlfC is less active on disaccharides with other linkages, we performed molecular docking analysis with various ligands that AlfC recognizes (fucose, 4NP-fuc, Fucα(1,6)GlcNAc, Fucα(1,2)Gal, Fucα(1,3)GlcNAc and Fucα(1,4)GlcNAc). The docking was reliable, faithfully reproducing the binding modalities observed in crystal structures in every case where they were available (AlfC-fucose, AlfC-4NP-fuc, AlfC-Fucα(1,6)GlcNAc; Fig. 5c–e), and increasing confidence in the docking results of ligands that AlfC processes with lower efficiency for which structures do not exist (Fucα(1,2)Gal, Fucα(1,3)GlcNAc, Fucα(1,4)GlcNAc; Fig. 5f, g). These results demonstrate that the other linkages do not place the second sugar in the aromatic pocket adjacent to the active site. Instead, these linkages place the second sugar closer to the D242 loop, where they might clash with the loop should a closed conformation be required for AlfC catalysis, as it is for other α-fucosidases. Together, these results provide the molecular basis for AlfC specificity on α(1,6)-linked disaccharides and *N-*glycans, and the requirement for endoglycosidase pre-treatment of IgG.

### Transfucosylation by other AlfC mutants

**Fig. 6:**
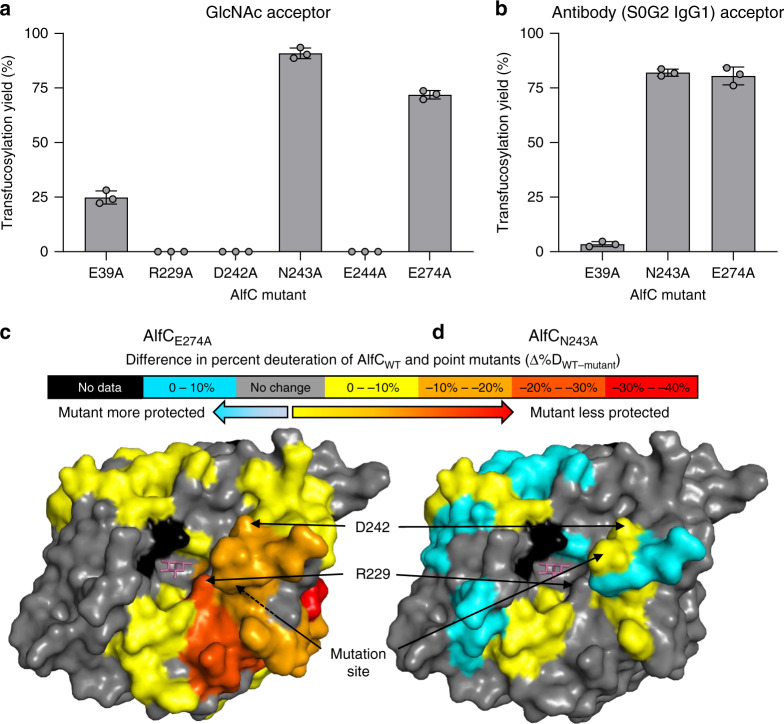
Analysis of AlfC transfucosylation mutants. **a** AlfC catalyzed transfer of fucose from α-fucosyl fluoride; and **b** IgG1 antibody (S0G2 glycoform). Separate reactions were measured with error bars representing standard deviation of *n* = 3 independent experiments. Data are presented as mean values ± SEM. **c**, **d** Hydrogen–deuterium exchange mass spectrometry analysis of transfucosylation mutants. Maximum differences in percent deuteration of peptides at 10 s or 1 min from **c** AlfC_E274A_ and **d** AlfC_N243A_ compared to wild-type. Differences are mapped onto a surface representation of AlfC bound to fucose (magenta). The site of mutation and the position of D242 are annotated with arrows. Source data are provided as a Source Data file.

The large decrease in turnover by several mutants suggests that multiple residues have key roles in the catalytic cycle. We wondered if any of these other mutants would have transfucosylation properties akin to E274A. We evaluated several mutants with low hydrolytic activity (E39A, R229A, D242A, N243A, E244A, E274A) for their ability to catalyze the transfer of fucose from α-fucosyl fluoride to GlcNAc (Fig. 6a). R229A, D242A, and D244A showed no activity, while E39A showed low activity. In contrast, N243A showed very high activity (~90% yield with respect to the acceptor GlcNAc), equal to or exceeding the yield of E274A. We then tested if E39A or N243A would have the ability to catalyze the transfer of fucose using a fully glycosylated antibody as the acceptor (Fig. 6b). E39A produced very low yields (~5%), while N243A again equaled the yields (~80%) provided by E274A. The resulting core fucosylated antibody carrying the full-length *N*-glycan was characterized using MALDI-TOF-MS (Supplementary Fig. S8). The α(1,6)-fucosyl linkage generated by the new transfer mutant AlfC_N243A_ was further confirmed by successful digestion with wild-type EndoS2 and AlfC (Supplementary Fig. S8). These transfucosylation results are surprising in light of the structures and kinetic experiments, as it is unlikely that either of these residues acts directly as the acid/base. These results suggest that these mutants favor transfucosylation through changes in enzyme structure and/or dynamics and that several mutations can produce similar results. They also suggest that there is nothing inherently unique about the ability of E274A to catalyze the fucosylation of fully glycosylated antibodies. This appears to be a general property for all AlfC fucosidase transfer mutants, despite the incapability of any AlfC enzyme (wild-type or mutant) to hydrolyze fucose from fully glycosylated antibodies (Fig. 1).

### Structure and dynamics of AlfC transfucosylation mutants

The paradoxical behavior of AlfC_E274A_ and AlfC_N243A_ led us to probe the structure and dynamics of these enzymes in order to understand their mechanisms as efficient transfucosidase mutants. We solved the X-ray crystal structures of both of these enzymes (Supplementary Table S2). They exhibited no major conformational changes compared to AlfC_WT_, with C_α_RMSD < 0.25 Å for each of the enzymes (Supplementary Fig. S9). Only AlfC_E274A_ showed a subtle change near the site of mutation, with a displacement of the W198 side chain ~4 Å due to an alternate rotamer conformation in order to occupy the cavity left by E274A (Supplementary Fig. S9). AlfC_N243A_ was nearly identical to wild-type, including at the site of mutation (Supplementary Fig. S9). This is not surprising, as N243 does not form contacts with any other residues in the AlfC crystal structures. Together, these results suggest that alanine mutations at AlfC positions 274 and 243 switch their functions from fucosidases to transfucosidases through changes in their conformational dynamics rather than through major rearrangements in overall protein folds, such as the unfolding of secondary structural elements.

Because our crystallographic data provide only an averaged structure, we sought to experimentally probe changes in the dynamics of the mutant enzymes. To do this, we performed hydrogen–deuterium exchange mass spectrometry analysis (HDX-MS). HDX-MS relies on the exchange of hydrogen with deuterium on peptide backbone amides and provides information on backbone solvent accessibility and dynamics, as residues that are more frequently exposed to solvent will undergo faster deuteration. By subtracting the percent deuteration for wild-type and mutant proteins for individual proteolytic peptides, we determined which regions of each protein display statistically significant changes in deuteration as a result of point mutation (Fig. 6c, d and Supplementary S10). In the wild-type enzyme and point mutants (E274A and N243A), deuterium uptake of peptides including E274 is low (~15% at 1 min) and unchanged by mutation, suggesting that the β-strand containing this residue is stable in solution for both the wild-type enzyme and point mutants (Supplementary Fig. S10). In contrast, peptides containing D242 have high deuterium uptake (~75% at 1 min) in the wild-type enzyme, consistent with this residue’s position on a mobile loop. The point mutant AlfC_E274A_ is markedly more dynamic and solvent accessible, particularly in the regions surrounding R229 and D242 (Fig. 6c). These residues interact to mediate open and closed conformations in MD simulations, with these results consistent with AlfC_E274A_ sampling more open conformations (Figs. 3d and 6c). The marked perturbation in R229 provides a potential rationale for the azide rescue seen with AlfC_E274A_, if azide ions occupy the E274A cavity to stabilize R229 and promote closed conformations. The differences in dynamics for AlfC_N243A_ were more subtle, with this enzyme displaying increases in deuteration in some regions, and decreases in others (Fig. 6d), both less pronounced than in AlfC_E274A_. However, like in AlfC_E274A_, the region containing D242 showed increases in deuteration, suggesting that this enzyme might also sample more open conformations.

### Prediction of new transfucosidase mutants

**Fig. 7:**
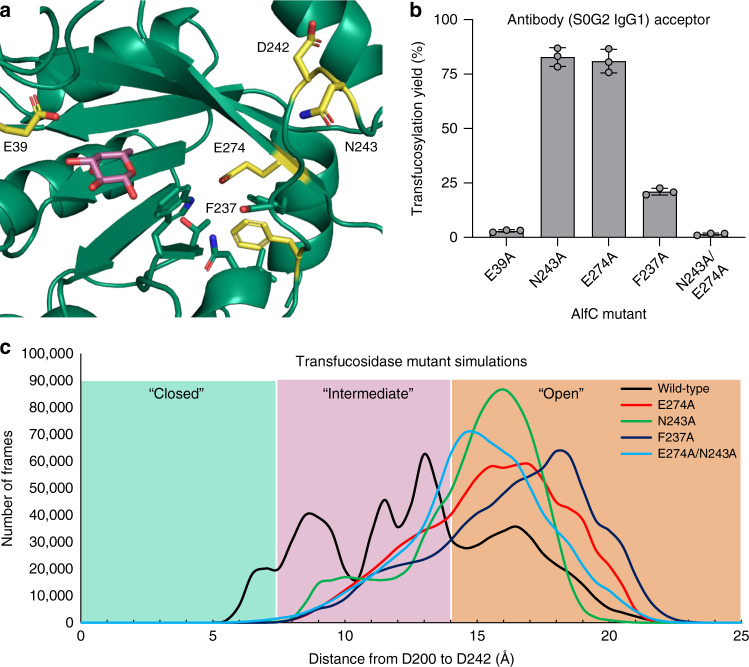
Structure and activity of fucosyltransferase mutants. **a** Cartoon representation of AlfC-fucose, highlighting the position of F237A in a hydrophobic pocket. Other fucosyltransferase mutations are also shown in yellow. **b** Fucosyltransferase activity of AlfC_F237A_ and AlfC_N243A/E274A_ using a fully glycosylated antibody as the acceptor, compared to E39A, N243A, and E274A (Fig. 6b). AlfC_F237A_ and AlfC_N243A/E274A_ samples were measured on separate reactions with error bars representing standard deviation of *n* = 3 independent experiments. Data are presented as mean values ± SEM. **c** Molecular dynamic simulation of AlfC transfucosylation mutants with frames plotted by the distance separating AlfC D200 and D242 O_δ_. Source data are provided as a Source Data file.

The HDX-MS analysis suggests that AlfC_E274A_ and AlfC_N243A_ are transfucosidase mutants that work by shifting the dynamic equilibrium of AlfC toward a more open conformation. This conformation would reduce hydrolytic activity, but still allow transfucosylation activity if acceptor substrate binding promotes a return to the closed conformation, suggesting that other mutations, which promote an open conformation could also act as transfucosidases. To test this, we mutated F237, which immediately precedes the acid/base loop, and inserts into the β-barrel, which we predicted would help the enzyme stabilize closed conformations (Fig. 7a). As expected, mutation of this residue to alanine resulted in transfucosylation activity, albeit with somewhat lower efficiency (~20%) than by AlfC_E274A_ and AlfC_N243A_, suggesting a generalizable mechanism (Fig. 7b). We then created the double mutant AlfC_E274A/N243A_ to see if the mutations would compound to create additive/synergistic effects. However, the double mutant lost nearly all of its transfucosylation activity, suggesting that AlfC conformational dynamics may need to be narrowly tuned to produce optimal fucosyl transfer.

To test our hypothesis that AlfC transfucosylation mutants sample more open conformations than their hydrolytic counterparts, we performed molecular dynamic simulations of each of the transfucosylation mutants and plotted the distance between D200 and D242 (Fig. 7c). As expected, these mutants, including the rationally-designed mutant F237A, almost exclusively sample intermediate and open conformations. The mean distance between D200 and D242 increased 2–4 Å in the transfucosylation mutants. In contrast, the mean distance between D200 and E39/E274 changed less than 1 Å, suggesting that the dynamic changes in transfucosylation mutants primarily involve D242 (Supplementary Fig. S11). Additionally, we performed a control simulation on the mutant N253A, which has hydrolytic activity equivalent to the wild-type enzyme (Fig. 4a). As expected, this enzyme sampled a mixture of open and closed conformations that resembled the wild-type enzyme (Supplementary Fig. S12), supporting the validity of our MD simulations. Finally, the double mutant AlfC_E274A/N243A_ that loses both hydrolytic activity and transfucosylation activity had an overall distance profile similar to the transfucosylation mutants, suggesting simulations in the apo state do not fully capture the complexity of an enzyme’s dynamic changes and its response to the substrate. Overall, however, these results show that transfucosylation mutants can be designed rationally by shifting enzyme dynamics toward open conformations, but that care must be taken to not disturb the enzyme too profoundly.

## Discussion

The important contributions of fucose to many biological processes, including antibody effector functions, demands our understanding of fucose-modifying enzymes, with which we will be able to specifically detect, modify, and control fucosylation to improve human health. We have provided a detailed structural and dynamic analysis of AlfC_WT_ and several highly efficient transfucosidase mutants. Our results provide the structural basis for α(1,6)-specificity of AlfC, identifying an aromatic +1 subsite adjacent to the active site that specifically accommodates GlcNAc in α(1,6)-linkages. They also provide a rationale for the requirement of endoglycosidase pre-treatment of antibodies before defucosylation, as additional sugars attached to the +1 sugar would clash with the enzyme surface. Understanding how the +1 subsite controls substrate recognition in other GH29A fucosidases might yield strategies for identifying fucosidases or fucosidase mutants capable of catalyzing specific reactions, including the defucosylation of fully branched IgG. Such an enzyme would be extremely desirable, as afucosylated antibodies bind Fcγ receptors more tightly, producing improved effector functions and clinical outcomes^29,30^. Several afucosylated monoclonal antibodies are now FDA-approved and represent the next generation of therapeutics for various cancers and inflammatory disorders^31^.

Our results suggest that, for hydrolysis, AlfC employs several catalytic residues, including a nucleophile (D200) and general acid/base. Structural homology and MD simulations suggest that D242 is the acid/base, which resides on a loop that adopts open and closed conformations. This is consistent with the mechanism of every α-fucosidase described to date for which structural data exists^7,8,18,20,23,26^. However, we could not conclusively determine that this is the acid/base in AlfC, as we were unable to obtain a crystal structure of AlfC in a closed conformation. This is not surprising, as our MD simulations suggest that AlfC only rarely samples the closed state, although, the computational expense of our comprehensive approach to analyzing the many AlfC mutants by this approach necessitated limited sampling and further sampling may be required to reach conformational equilibrium in every case. Our determination of the acid/base is compounded by azide rescue experiments that gave conflicting and structurally implausible results. Azide only rescued the E39A mutant when using 4NP-fuc as a substrate, while it only rescued the E274A mutant when using α-fucosyl fluoride. Our crystal structures, HDX-MS analyses, and MD simulations provide no plausible mechanism by which either of these residues could act directly as the acid/base. Both residues are greater than 9 Å from the nucleophile, have no access to the reactive C1 of fucose, and are located on stable secondary structure elements that remain unchanged during simulations. While azide rescue has long been employed as a key method for identifying the acid/base residue in glycosidases^27^, its use in α-fucosidases has produced perplexing results in several other instances. In Ssα-fuc and NcFuc, the sequence equivalent of E39 was rescued by azide, however, isolation of the expected rescue product was unsuccessful in Ssα-fuc, and not attempted in NcFuc^6,9^, mirroring our results with AlfC_E39A_. In FucA1 and cFase I, the sequence equivalent of AlfC E274 was rescued by azide^10,25^, and in FucA1, the expected rescue product could even be isolated, again mirroring our results with AlfC. However, none of these enzymes had structural data to support the assignment of acid/base. In FucA1, homology modeling predicted that the acid/base residue lies in the same position as AlfC E274, which the authors noted was a problem^10^. It remained plausible that the modeling was inaccurate, but now we have crystal structures confirming conservation of structure in an enzyme that demonstrates azide rescue of an unexpected residue. It is possible that AlfC undergoes deep conformational changes or employs a proton relay from a distant acid/base residue, but this is directly refuted by HDX-MS, MD simulations, and crystallographic data showing these residues on immobile secondary structure elements. Instead, it is more likely that AlfC employs a structurally conserved mechanism using D242 as the acid/base, and that azide rescue may be an imperfect method for determining the acid/base residue in some α-fucosidases that require substantial conformational changes for activity. Azide rescues acid/base mutants by filling cavities left by truncated side chains and replacing their role. It is possible that azide rescued E39 and E274 mutants by promoting conformational changes required for catalysis, such as the adoption of closed conformations. Indeed, HDX-MS of AlfC_E274A_ showed that this point mutation markedly destabilizes the active site. The fact that azide rescue was not observed for D242 mutations may be because AlfC preferentially adopts open conformations, so there is no cavity to fill when mutating D242. Such a cavity would exist in the closed state, but homology, MD simulations, and mutagenesis suggest that the closed state relies on a salt bridge between D242 and R229, and therefore may not exist in D242 mutants. Resolving the structure/azide rescue paradox of some α-fucosidases will require structures of other enzymes that appear by azide rescue to employ distant acid/base residues (e.g., Ssα-fuc, NcFuc, FucA1, cFase I). Structures of these enzymes in catalytically active states will be paramount, and methods such as cryo-EM might prove valuable in capturing low-population states.

Our prediction of open and closed states with respect to D242 yielded a successful strategy for the rational creation of new transfucosidase mutants. HDX-MS and MD simulations suggest that several mutations (e.g., E274A, N243A) shift the dynamic equilibrium toward open conformations, thereby reducing hydrolysis. To understand how these mutations produce transfucosylation, one might draw inspiration from the mechanisms of non-Leloir transglycosylases^32^. These are unusual glycoside hydrolases that naturally catalyze transglycosylation rather than hydrolysis. Once a covalent enzyme-intermediate has formed, how do these enzymes preferentially select sugars as acceptors, even when water is millions of times more abundant and equally capable of acting as an acceptor? It is thought that these enzymes possess an aromatic +1 subsite adjacent to the active site in which the acceptor sugar binds. This binding leads to subtle but important conformational changes in the active site that lowers the activation energy for transglycosylation^32^. Meanwhile, hydrolysis does not occur because water is incapable of causing the conformational changes needed for catalysis. If we apply these general principles to AlfC, we might propose that GlcNAc binding in the +1 subsite promotes a closed conformation, while water does not. Therefore, if a mutation shifts the equilibrium toward open conformations, the enzyme only becomes catalytically competent when GlcNAc binds, thereby increasing the ratio of transglycosylation to hydrolysis. Although we lack direct evidence for this mechanism, we have shown that a mutation designed to promote open conformations (F237A) produces transfucosylation activity. These results are not without precedent; directed evolution experiments with Tmα-fuc identified three mutations (T264A, Y267F, L322P) that, when combined, increased its transfucosylation efficiency 32-fold^21^. Strikingly, these mutations are very similar to the transfucosylation mutants identified in this paper: in crystal structures, Tmα-fuc Y267 is the structural analog of AlfC N243; Tmα-fuc L322 is directly adjacent to AlfC E274; and Tmα-fuc T264 is directly adjacent to AlfC F237. Four of these residues (T264, L322, F237, and E274) are involved in stabilizing the β-barrel adjacent to the mobile loop which adopts open and closed conformations. The other two residues (Y267, N243) lie directly on this loop, and would plausibly interact with the substrate during a closed conformation. Therefore, all of these mutations might be expected to shift the equilibrium toward open conformations. Notably, when several of these mutations are combined in AlfC, the enzyme loses its transfucosylation capabilities, suggesting that it might be possible to shift the equilibrium too far (e.g., AlfC_E274A/N243A_ might be incapable of returning to closed conformations in response to GlcNAc binding). Alpha-fucosidase acid/base loops have previously been subjected to engineering; in one study, a segment of the loop was substituted from an efficient transfucosidase (*Cp*Afc2) to an inefficient one (*Bb*AfcB) to make the latter more efficient^33^. Our results with AlfC suggest that the dynamics of the acid/base loop are sensitive to mutation and modulate transfucosylation efficiency. It may be that seemingly disparate approaches with AlfC, Tmα-fuc and *Bb*AfcB share a common underlying mechanism that would provide a path forward for the rational discovery of transfucosidase mutants in other α-fucosidases.

The paradox that AlfC transfucosylation mutants can fucosylate fully branched glycosylated antibodies to create a product that neither the wild-type enzyme nor any point mutant can hydrolyze is perplexing. The easiest explanation for this phenomenon would be if transfucosylation mutants added fucose to antibodies in a linkage or site that is non-hydrolyzable by AlfC (e.g., in an α(1,3) linkage, or directly to a protein side chain). However, we carefully excluded this possibility in our original characterization of AlfC_E274A_, confirming by mass spectrometry and NMR that fucose is added solely to the first GlcNAc in an α(1,6)-linkage^12^. Furthermore, in our studies of AlfC_N243A_ presented here, we showed that the fucose that gets added can be hydrolyzed by AlfC_WT_ following treatment with EndoS2. Alternative explanations are less readily apparent; perhaps only the more open conformations that promote transfucosylation are large enough to accommodate a fully branched glycan. One must explore whether transfucosidase mutants of other enzymes are capable of creating non-hydrolyzable products using large acceptors. Understanding how these enzymes create products they cannot hydrolyze may reveal a strategy for creating extremely efficient glycosyltransferases.

## Methods

### Cloning, expression, and purification

Wild-type AlfC was cloned into the PET22b-CPD vector as previously described^12^. The plasmid was transformed into *E. coli* BL21(DE3)pLysS and expressed in LB medium overnight at 18 °C after induction with 0.5 M IPTG at an OD_600_ of 0.6. Cells were harvested (5000 × *g* for 20 min) and then pellets were resuspended in 500 mM NaCl, 15 mM imidazole, 50 mM Tris-HCl pH 7.4 (Buffer A) and lysed by four freeze/thaw cycles, then 30 min incubation with lysozyme supplemented to 100 µg/ml and benzonase nuclease (Sigma). Cleared lysate was passed over His-Pur NiNTA columns (ThermoScientific), washed with 20 column volumes of Buffer A, then ten column volumes of 150 mM NaCl, 50 mM Tris pH 7.4 (Buffer B). Protein was then eluted by incubating the columns overnight at 4 °C in Buffer B supplemented with 100 µM phytic acid, which induces the self-cleavage of the CPD-His_10_ domain. For crystallization experiments, AlfC was buffer exchanged into 100 mM NaCl, 20 mM Tris pH 7.4 (Buffer C), and further purified by size-exclusion chromatography in a Superdex 200 10/300 GL column (GE Healthcare). For hydrogen–deuterium exchange experiments, AlfC was then buffer exchanged into PBS. AlfC point mutants were generated by PCR-based site-directed mutagenesis, and sequences were confirmed by Genewiz (https://www.genewiz.com). For kinetic experiments, proteins were purified using fully disposable consumables to prevent the possibility of cross-contamination. Size-exclusion chromatography was not performed, so all enzymes were assessed for proper folding by measuring melting temperatures using differential scanning fluorimetry, as previously described^34^. All enzymes were assessed to be >90% pure by SDS/PAGE.

### Crystallization and data collection

Wild-type AlfC was concentrated to 20 mg/ml, and 250 nl of protein was combined with 250 nl of mother liquor (18% [w/v] polyethylene glycol 3350, 0.1 M bis-tris propane pH 7, 20 mM Na_2_H/KH_2_ phosphate, 1% [v/v] glycerol) in sitting drops, with crystals appearing after 5 days. These unliganded crystals were harvested in mother liquor supplemented with 20% (v/v) glycerol for cryoprotection, and flash cooled in liquid nitrogen. AlfC-fucose crystals were obtained by soaking apo crystals in cryoprotectant supplemented with 10 mM fucose for ~1 min before being flash cooled. AlfC_E274A_ crystals were obtained by the same method as AlfC_WT_. AlfC_D200A_-4NP-fuc and AlfC_N243A_ crystals were grown using the same method as AlfC_WT_, except they were soaked for several minutes in cryoprotectant supplemented with 5 mM 4NP-Fuc before being flash cooled. AlfC_D200A_-Fucα(1,6)GlcNAc crystals were grown by the same method as AlfC_D200A_-4NP-fuc, except the apo crystals were soaked in cryoprotectant supplemented with 10 mM Fucα(1,6)GlcNAc (MuseChem). AlfC crystals grew in several distinct, but closely related space groups (C222_1_, P2_1_2_1_2_1,_ and P2_1_ [twinned]) and morphologies (rectangular plates, rhomboidal, and pentagonal plates) depending on small batch-to-batch variability in mother liquor/protein preparation. Data for AlfC_WT_ and AlfC_N243A_ were collected at the Advanced Photon Source (APS) beamline 23-ID-B on a Dectris Pilatus3 6M detector. Data for AlfC_E274A_ were collected at BNL National Synchrotron Light Source II beamline 17-ID-1 (AMX) on an Eiger 9M detector. Data for AlfC_D200A_-Fucα(1,6)GlcNAc were collected at BNL National Synchrotron Light Source II beamline 17-ID-2 (FMX) on an Eiger 16M detector. Data for AlfC_D200A_-4NP-fuc were collected at the Stanford Synchrotron Radiation Lightsource beamline 12-2 on a Dectris Pilatus3 6M detector.

### Structure determination, refinement, and analysis

The unliganded-AlfC structure was solved first, using the GH domain from *Bt*Fuc2970 (PDB 4PCT) as a search model for molecular replacement using MOLREP^35^. Models were further built and refined using Coot^36^ and REFMAC^37^, respectively. Subsequent models were solved using unliganded-AlfC as a search model for molecular replacement, and further refined using the same methods. AlfC_N243A_ crystals were likely twinned according to Xtriage^38^ (multivariate *Z*-score *L*-test = 9.9), so they were refined using intensity-based twin refinement in REFMAC. Structural homologs were searched for using the Dali web server^17^, and graphics were created in PyMOL. Ligands were validated by generating polder maps in Phenix^38^ (Supplementary Fig. S13).

### Mass spectrometry analysis of antibody defucosylation

Activity on fully glycosylated Rituximab was measured using 5 µM Rituximab with 1 µM AlfC_WT_ or AlfC_E274A_ in PBS for 1 month at 25 °C. Activity on partially deglycosylated Rituximab was measured using 5 µM Rituximab with 50 nM EndoS2 and 1 µM AlfC_WT_ in PBS overnight at 25 °C. EndoS2 rapidly hydrolyzes antibody glycans between the first and second *N*-acetylglucosamine residues, leaving one *N-*acetylglucosamine residue and fucose attached to each heavy chain of the antibody. After the designated incubation, 10 μl aliquots of each reactions were quenched by the addition of 1.1 μl trifluoroacetic acid. The quenched reactions were then mixed with 50 mM TCEP, and analyzed by LC–MS using an Accela LC System attached to an LXQ linear ion trap mass spectrometer (ThermoScientific, Waltham, MA), as previously described^39^. Relative amounts of substrate and hydrolysis products were quantified after deconvolution of the raw data using BioWorks (ThermoScientific, Waltham, MA). Relative intensities by mass were then exported to GraphPad Prism and replotted, overlayed, and annotated.

### Kinetic analyses with 4NP-Fuc

To determine an appropriate buffer, the pH dependence of AlfC was explored using the PCB buffer system. AlfC activity was measured using 50 µl reactions with 1 mM 4NP-Fuc and 100 nM AlfC in 100 mM PCB buffer (sodium propionate, sodium cacodyolate, bis-tris propane; Qiagen) between pH 4 and 8. Reactions proceeded for 20 min at 25 °C and we then quenched by addition of 50 µl 1 M ice-cold sodium carbonate pH 11, and absorbance at 420 nm was measured using a SpectraMax iD3 plate reader in 96 well flat-bottom plates (Sigma), with no-enzyme controls subtracted. AlfC exhibited a bell-shaped curve with maximal activity around pH 5–6, so 100 mM sodium citrate pH 5.8 was used for further experiments (Supplementary Fig. S14). For initial azide rescue experiments, the activity of AlfC_WT_ and point mutants was tested by the same method above, except in the presence and absence of 200 mM sodium azide. Full kinetic analyses were performed for select enzymes displaying very high or very low activity, as follows: 50–2000 nM AlfC was incubated for 30–300 min with 62.5 μM–12.5 mM 4NP-Fuc in 100 mM sodium citrate pH 5.8 at 25 °C in a total volume of 50 µl, and then quenched with 50 µl 1 M ice-cold sodium carbonate pH 11. Enzyme concentrations and reaction times were selected so that product formation was linear with respect to time, and fell within the linear range of detection with a standard curve made with 4-nitrophenol. The molar absorptivity of 4-nitrophenol was calculated to be 1.625 mM^−1^ using this method. Kinetic parameters (*k*_cat_ and *K*_M_) were determined by plotting *V*_0_ versus substrate concentration, and fit to a Michaelis-Menton curve using GraphPad Prism.

### Azide rescue experiments monitored by ^19^F NMR

AlfC mutants (1 μM) were added to a mixture of α-fucosyl fluoride^12^ (7.5 mM) and NaN_3_ (200 mM) in a buffer (PBS, 150 mM, pH 7.4, 400 μl) containing TFA (1 μl) as the internal standard for ^19^F NMR. The reaction mixture was incubated at room temperature and monitored with proton decoupled ^19^F NMR during the time course. A control reaction without any NaN_3_ was carried out side-by-side to eliminate the effect of spontaneous and enzymatic hydrolysis of α-fucosyl fluoride. Reaction rates of azide rescue experiments with different AlfC mutants were calculated according to the ^19^F signal decrease of the donor substrate (*δ* = 152.59 ppm), α-fucosyl fluoride, during the time course using TFA as the internal standard (*δ* = 76.55 ppm). To prepare the product of azide rescue reaction, AlfCE274A (1 μM) was added to a mixture of α-fucosyl fluoride (5 mg, 10 mg/ml) and NaN_3_ (200 mM) in a buffer (PBS, 150 mM, pH 7.4, 500 μl). The reaction mixture was incubated at room temperature and monitored with TLC. After the reaction completed, the rescue product was purified with flash gel chromatography by linear elution (ethyl acetate/methanol/water, 17:2:1) to obtain azide rescue product (4.9 mg, 86%) as a white powder. ^1^H NMR (D_2_O, 400 MHz): *δ* = 5.02 (d, *J*_1,2_ = 4.0 Hz, 1H, H-1), 3.82 (m, 1H, H-5), 3.72 (m, 1H, H-3), 3.61 (m, 1H, H-2), 3.41 (m, 1H, H-4), 1.21 (d, *J* = 6.4 Hz, 3H, -CH_3_). ^13^C NMR (D_2_O + 1% DMSO, 100 MHz): *δ* = 90.99 (C-1), 73.61 (C-3), 73.33 (C-4), 71.65 (C-5), 70.57 (C-2), 15.86 (-CH_3_). ESI-MS: calculated for azide rescue product, *M* = 189.07 Da; found (*m*/*z*), 190.01 [M+H]^+^.

### Chemoenzymatic fucosylation

The enzymatic fucosylation of GlcNAc and antibody was performed following the previously published method^12^. For fucosylation of GlcNAc, AlfC mutants (25 µg, 0.5 mg/ml) were added to a mixture of synthetic donor substrate α-fucosyl fluoride (0.5 mg, 3.0 µmol) and acceptor GlcNAc (0.44 mg, 2.0 µmol) in a buffer (PBS, 150 mM, pH 7.4, 50 µl), and the reaction mixture was incubated at 37 °C for 5 min. The crude product was purified with a P2 size-exclusion column (Bio-Rad) and then lyophilized to give a pure fucosylated product. For antibody core fucosylation, AlfC mutants (20 µg, 2 mg/ml) were added to a mixture of α-fucosyl fluoride (9.6 µg, 0.056 µmol) and glycoengineered homogeneous S0G2-Herceptin (200 µg, 0.007 µmol) in a buffer (PBS, 150 mM, pH 7.4, 10 µl). The solution was incubated at 37 °C for 8 h and monitored with MALDI-TOF-MS analysis (Bruker). The crude transfer product was purified with a protein A column (GE Healthcare) and promptly dialyzed against a buffer (PBS, 150 mM, pH 7.4) at 4 °C to give the pure core fucosylated antibody. To characterize the core fucosylated antibody, immobilized EndoS2^40^ (0.1 mg/ml) and AlfC (0.5 mg/ml) were added to a solution (PBS, 150 mM, pH 7.4, 20 µl) containing the S0G2F-Herceptin (200 µg, 0.007 µmol). The reaction mixture was incubated at 30 °C for 12 h. The crude hydrolytic product was purified with a protein A column and dialyzed against a buffer (PBS, 150 mM, pH 7.4) at 4 °C to give the pure deglycosylated, defucosylated antibody.

### HDX-MS

The coverage maps for all constructs of AlfC were obtained from undeuterated controls as follows: 3 µl of 10 µM sample in PBS was diluted with 97 µl of ice-cold quench (50 mM Glycine, 1 M Guanidine-HCl, pH 2.4) prior to the injection into a Waters HDX nanoAcquity UPLC (Waters, Milford, MA) with in-line protease XIII/pepsin digestion (NovaBioAssays). Peptic fragments were trapped on an Acquity UPLC BEH C18 peptide trap and separated on an Acquity UPLC BEH C18 column. A 7 min, 5–35% acetonitrile (0.1% formic acid) gradient was used to elute peptides directly into a Waters Synapt G2-Si mass spectrometer (Waters, Milford, MA). MSE data were acquired with a 20–30 V ramp trap CE for high energy acquisition of product ions as well as continuous lock mass (Leu-Enk) for mass accuracy correction. Peptides were identified using the ProteinLynx Global Server 3.0.3 (PLGS) from Waters. Further filtering of 0.3 fragments per residues was applied in DynamX 3.0.

For each construct, the HD exchange reactions and controls were acquired using a LEAP autosampler controlled by Chronos software. The reactions were performed as follows: 3 µl of 10 µM AlfC was incubated in 47 µl of PBS, 99.99% D2O, pD 7.4. All reactions were performed at 25 °C. Prior to injection, deuteration reactions were quenched at various times (10 s, 1 min) with 100 µl of 100 mM Glycine buffer, 2 M Guanidine-HCl, pH 2.4. All deuteration time points were acquired in triplicates. Back exchange correction was performed against fully deuterated controls acquired by incubating 3 µl of 10 µM of protein in 27 µl PBS, 99.99% D2O, pD 7.4 containing 7 M deuterated Guanidine DCl for 2 h at 25 °C followed by the addition of 20 µl PBS, 99.99% D2O, pD 7.4 and incubation for an additional 1 h prior to quenching (without guanidine-HCl).

The deuterium uptake for all identified peptides with increasing deuteration time and for the fully deuterated control was determined using Water’s DynamX 3.0 software. The normalized percentage of deuterium uptake (%*D*_*t*_) at an incubation time *t* for a given peptide was calculated as follows: with *m*_*t*_ the centroid mass at incubation time *t*, *m*_0_ the centroid mass of the undeuterated control, and *m*_f_ the centroid mass of the fully deuterated control. Percent deuteration difference plots, Δ%*D*_*t*_(wild type – mutant), displaying the difference in percent deuteration between the wild type and each individual mutant construct for all identified peptides, at all deuterium incubation time probed were generated. Confidence intervals for the Δ%*D* plots were plotted lines and used to determined peptides with statistically significant differences in deuterium uptake between the wild-type and mutant enzymes.

### Sedimentation

All sedimentation measurements were carried out using a Beckman Coulter Optima XL-I analytical ultracentrifuge equipped with a 4-hole An-60Ti rotor. For sedimentation equilibrium measurements, AlfC protein samples prepared at three concentrations ranging from 3 to 11 μM in buffer containing 50 mM Tris-HCl, 50 mM NaCl at pH 7.4 were loaded into a cell equipped with a 6-hole epon charcoal-filled centerpiece. The protein was centrifuged at 20 °C at rotor speeds of 14, 17, and 20K rpm and concentration versus radius scans were acquired at 280 nm after 8 and 9 h of centrifugation at each speed. A step size of 0.001 cm with 5 averages per step was employed for each scan. Data were analyzed using the Heteroanalysis program^41^ (https://core.uconn.edu/resources/biophysics#au-software) to a single ideal species and dimer-tetramer models, with the former yielding better agreement with the data, based on the rmsd of the fit and the quality of the residuals of the fit. The partial specific volume and the solvent density values used in the analysis were calculated using the program Sednterp (http://rasmb.org/sednterp/).

Sedimentation velocity measurements were performed on AlfC protein prepared at 11 μM in the same buffer as that used for sedimentation equilibrium measurements. The sample was centrifuged at 42,000 rpm at 18 °C in a standard two-hole cell equipped with an epon charcoal-filled centerpiece. A total of 759 absorbance scans were acquired at 295 nm in continuous mode. The data (every other scan) were analyzed using SEDFIT^42^ to a continuous distribution of sedimentation coefficients c(s) model to generate the species distribution.

### Molecular dynamics

Tetrameric AlfC from the apo crystal structure (PDB 6O18) was modified using ModLoop^43^ to build in the disordered region (residues 248–263). Mutant AlfC structures and input files were created from the resulting wild-type tetramer structure using the CHARMM-GUI Glycan Reader & Modeler web application^44^. Input files were generated for the openMM packages^45^ utilizing the CHARMM 36 Additive Force Field^46^ with the TIP3P water model^47^. The molecule was solvated in a periodic water box containing 0.15 M KCl, with box boundaries buffering 1 nm away from the molecule. A 1 nm cutoff distance was used for the Lennard–Jones interaction calculations and particle-mesh Ewald, with an Ewald error tolerance of 0.0005 used for the calculation of long-range electrostatic interactions. A 2 fs time step was used for integration with temperature and pressure held constant at 298.15 K and 1 atm, respectively. Before the simulation run, the system energy was minimized and then equilibrated for 20 ps. The simulations were then run for 100 ns utilizing the CHARMM 36 m force field and results were visualized and processed using VMD^48^. Distances from each condition were obtained using the MDTraj python analysis package^49^.

### Molecular docking

Ligand structures for docking were created using the carbohydrate builder of the glycam web server (glycam.org). Docking was performed using AutoDock Vina using standard methods, with flexibility allowed in the ligand, but not the protein^50^. The correct solution was deemed to be the solution with the greatest binding energy that placed fucose in the active site (in most cases, this was the top solution overall).

### Reporting summary

Further information on research design is available in the Nature Research Reporting Summary linked to this article.

## Supplementary information

Supplementary Information

Peer Review File

Reporting summary

## Data Availability

The atomic coordinates have been deposited in the Protein Data Bank, with PDB ID codes: 6O18 (AlfCWT), 6O1A (AlfC-fucose), 6O1C (AlfC-4NP-fuc), 6O1I (AlfCE274A), 6O1J (AlfCN243A-fucose), 6OHE (AlfCD200A-Fucα1,6-GlcNAc). Source data are provided with this paper.

## Source data

Source Data
