# Supplementary material for: Structure and dynamics of an α-fucosidase reveal a mechanism for highly efficient IgG transfucosylation
Source: Nat Commun. 2020 Dec 4;11:6204. doi: 10.1038/s41467-020-20044-z (PMC7718225; doi:10.1038/s41467-020-20044-z)
Supplement: Supplementary file 1 — Supplementary Information [file 41467_2020_20044_MOESM1_ESM.pdf]

Structure and dynamics of an α-fucosidase reveal a mechanism for highly efficient IgG transfucosylation

Erik H. Klontz[1,2,3], Chao Li[4], Kyle Kihn[5], James K. Fields[1,2,3], Dorothy Beckett[4], Greg A. Snyder[1], Patrick L. Wintrode[5], Daniel Deredge[5], Lai-Xi Wang[4], Eric J. Sundberg[1,2,6,7*]

[1]Institute of Human Virology, [2]Department of Microbiology & Immunology, [3]Program in Molecular Microbiology & Immunology, University of Maryland School of Medicine, Baltimore, MD 21201 USA

[4]Department of Chemistry and Biochemistry, University of Maryland, College Park, MD 20742, USA

[5]Department of Pharmaceutical Sciences, University of Maryland School of Pharmacy, MD 21201 USA

[6]Department of Medicine, University of Maryland School of Medicine, Baltimore, MD 21201, USA

[7]Present address: Department of Biochemistry, Emory University School of Medicine, Atlanta, GA 30322, USA

Supplemental Figures

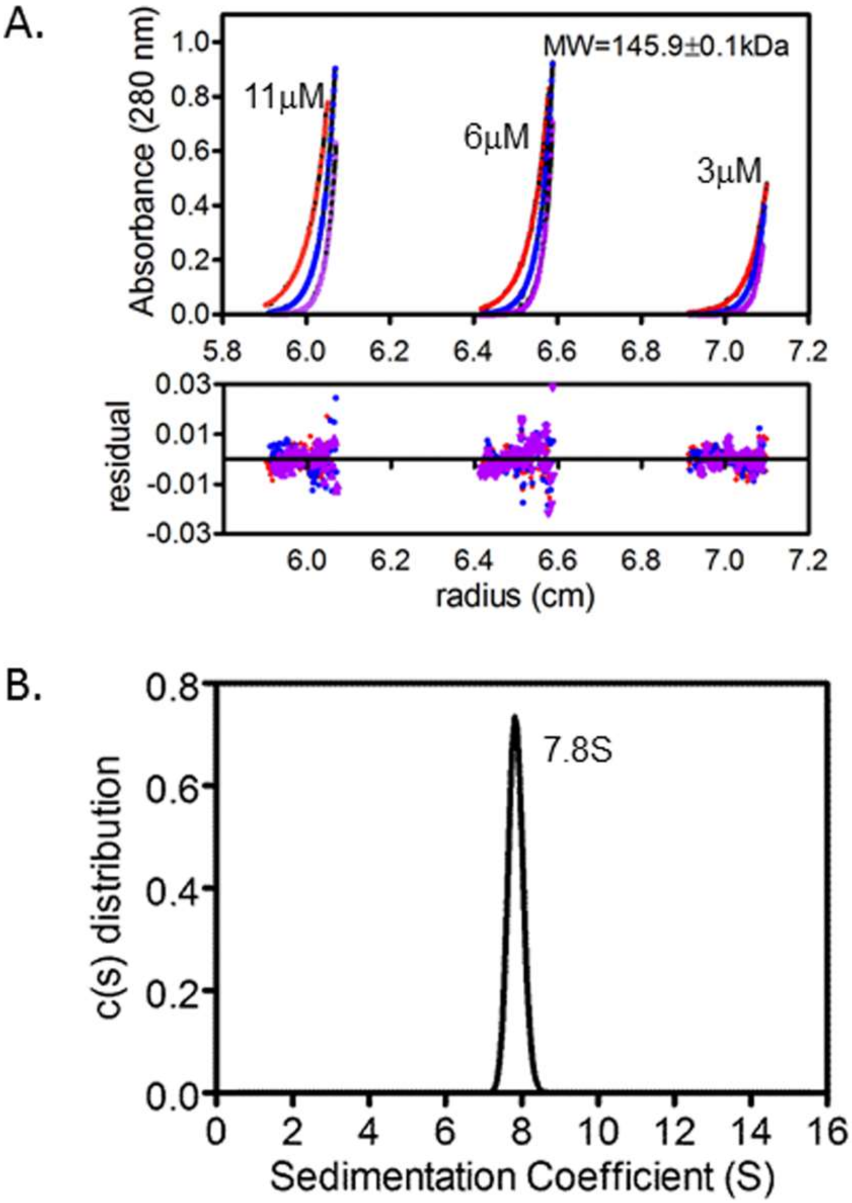

Supplementary Figure 1: Sedimentation analysis indicates that AlfC is a tetramer in solution. (A) Global analysis of sedimentation equilibrium data obtained at three loading concentrations and centrifuged at three rotor speeds indicates that the protein is well-described as a single species with an average molecular weight that is within 7% of the value expected for a tetramer (157 kDa). (B) Sedimentation velocity data analyzed using Sedfit yields a single species of 7.8S with a molecular weight of 144kDa (assuming a frictional ratio of 1.27).

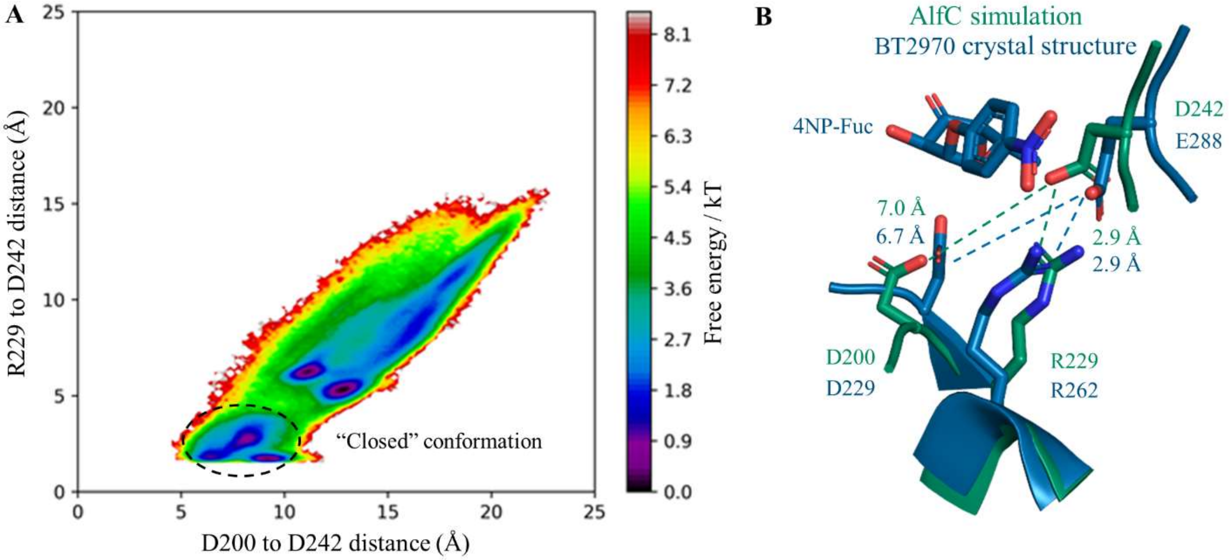

Supplementary Figure 2. AlfC$_{WT}$ simulation conformations of D242. **(A)** Wild-type AlfC active site free energy surface with respect to the distance between R229 and D242 and the distance between the nucleophile D200 to D242. Pairwise distances were measured from and/or to O_δ atoms (D200 and D242) and N_η (R229). Pairwise residue distance measurements returned four distances per monomer per frame. With 4 monomers and 52000 frames, a total of 840,000 distances were generated for each residue pairwise distance. Two-dimensional histograms were generated and free energy was calculated using -kTln P_{Dij}, where P_{Dij} is the probability for the measured distances to be within the bin ij, k is Boltzmann's constant, and T is temperature (298 K). The bin size is 0.083 Å for both coordinates. (B) The "closed" conformation of AlfC D242 from simulations closely resembles the catalytically active state of BT2970, including the distance and orientation of catalytic residues, as well a salt-bridge with an active-site arginine.

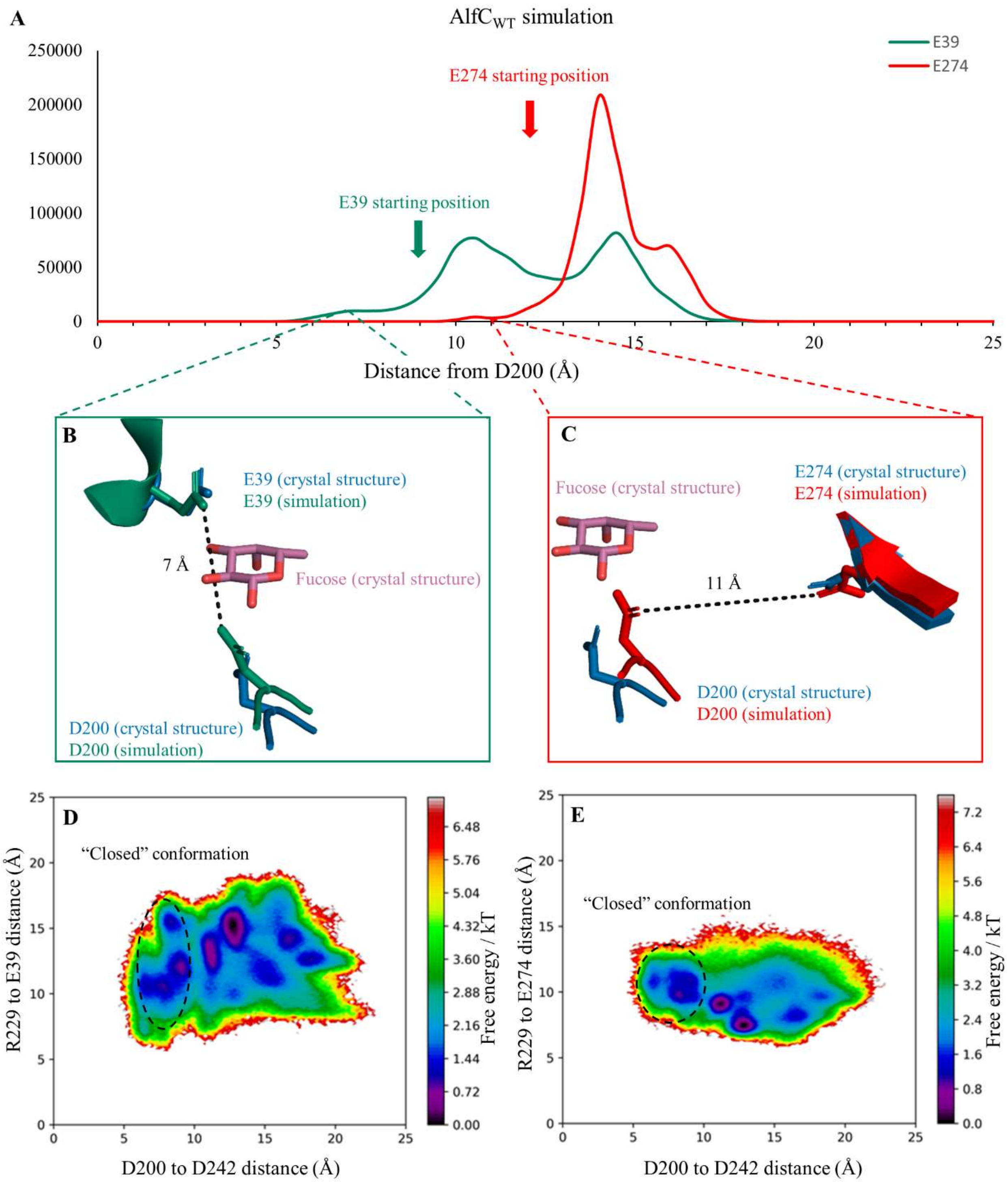

Supplementary Figure 3. AlfC$_{WT}$ simulation conformations of E39 and E274. **(A)** Residues E39 and E274 tended to drift away from D200 during simulations. **(B-C)** In rare frames where E39 and E274 were closer to D200 than their starting position, their orientations were similar to those seen in crystal structures and non-productive for catalysis. **(D-E)** Wild-type AlfC active site free energy surface with respect to the distance between R229 and E39 or E274 and the distance between the nucleophile D200 to D242. Pairwise distances were measured from and/or to O_δ atoms (D200 and D242), O_γ (E39 and E274) and N_η (R229). Pairwise residue measurements returned four distances per monomer per frame. With 4 monomers and 52000 frames, a total of 840,000 distances were generated for each residue pairwise distance. Two-dimensional histograms were generated and free energy was calculated using -kTln P_{Dij}, where P_{Dij} is the probability for the measured distances to be within the bin ij, k is the Boltzmann constant, and T is temperature (298 K). The bin size is 0.083 Å for both coordinates.

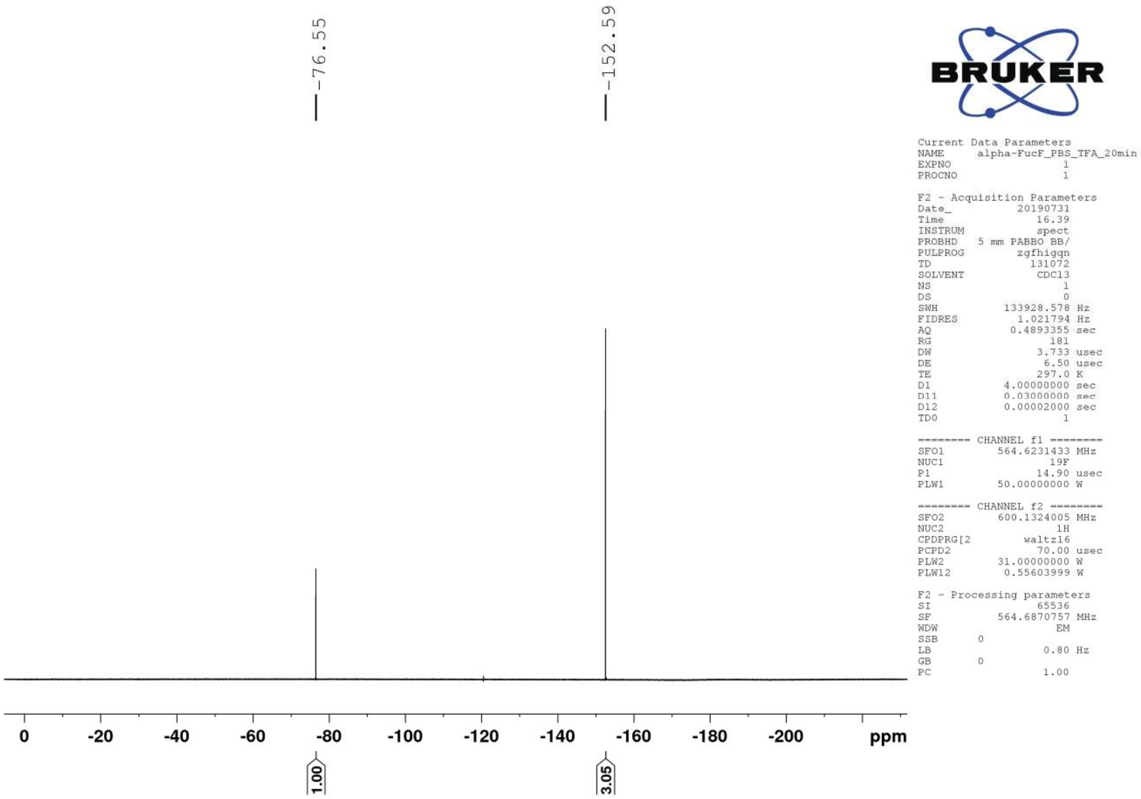

Supplementary Figure 4. ^{19}F NMR spectrum of an azide rescue reaction containing α-fucosyl fluoride, AlfC E274A and NaN_3 in a PBS buffer.

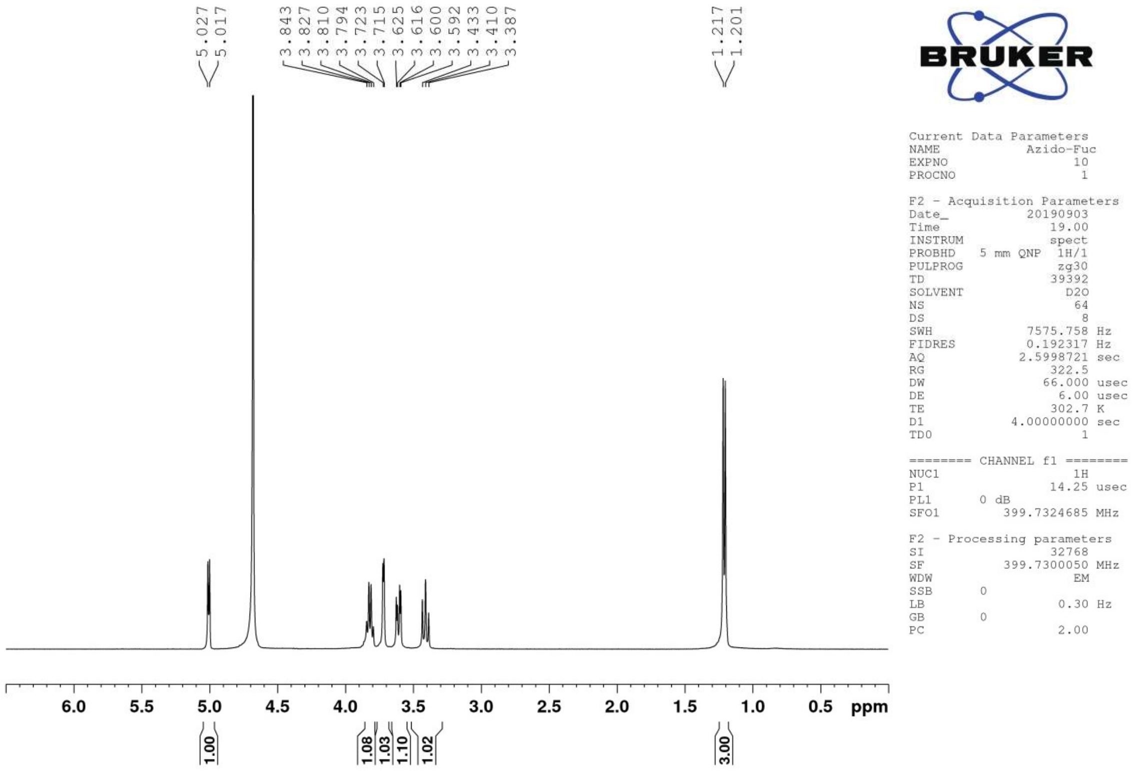

Supplementary Figure 5. ^{1}H NMR spectrum of α-fucosyl azide.

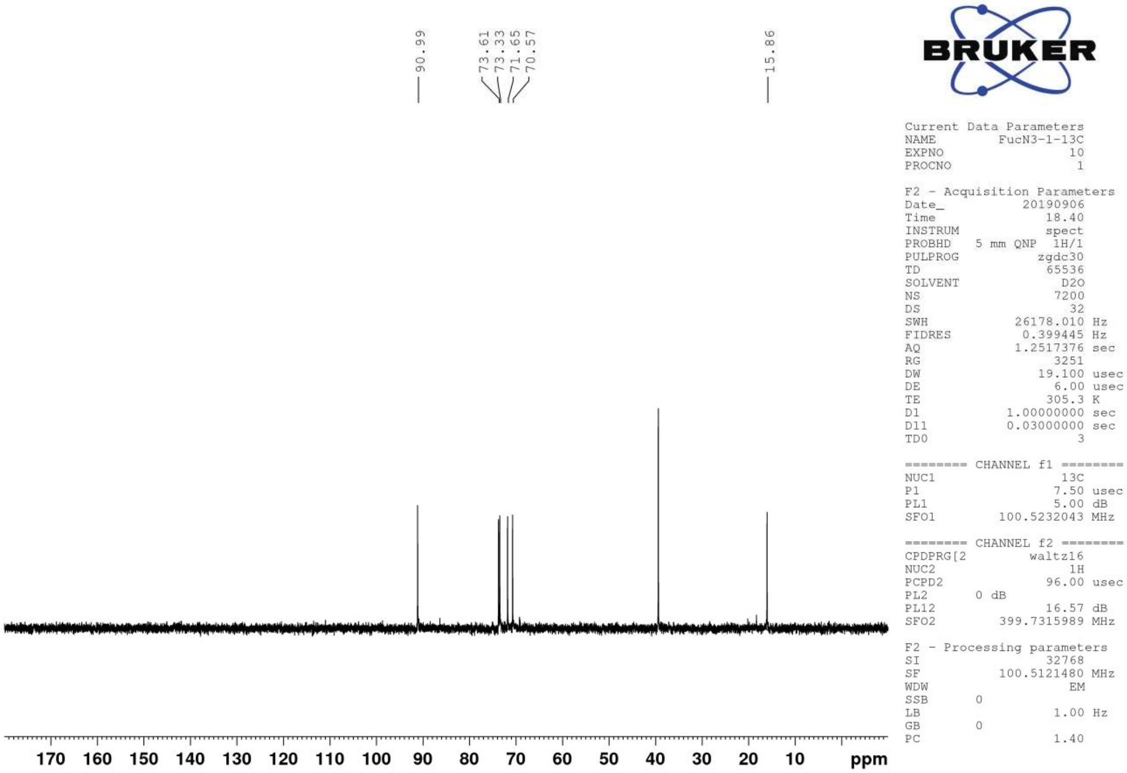

Supplementary Figure 6. ^{13}C NMR spectrum of α-fucosyl azide.

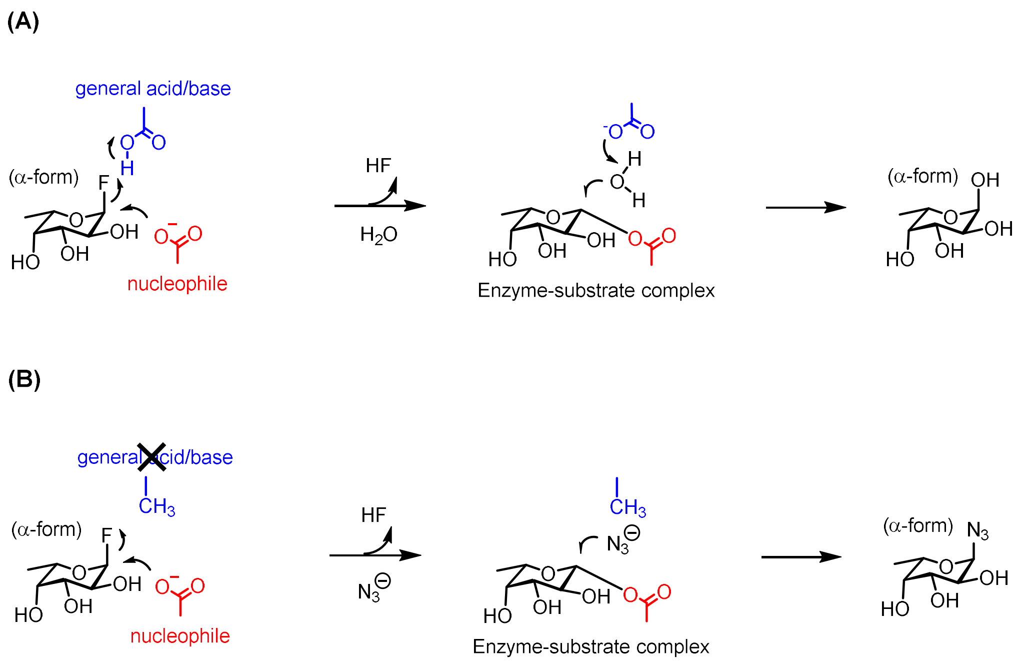

Supplementary Figure 7. Catalytic mechanisms of α-fucosyl fluoride using $AlfC_{WT}$ and $AlfC_{E274A}$. (A) Hydrolysis by AlfC α-fucosidase via double displacement. (B) Azide rescue of AlfC α-fucoligase mutant E274A.

A

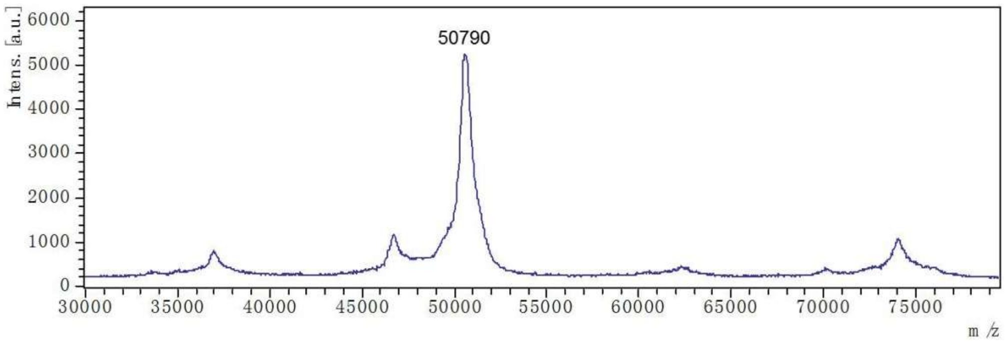

B

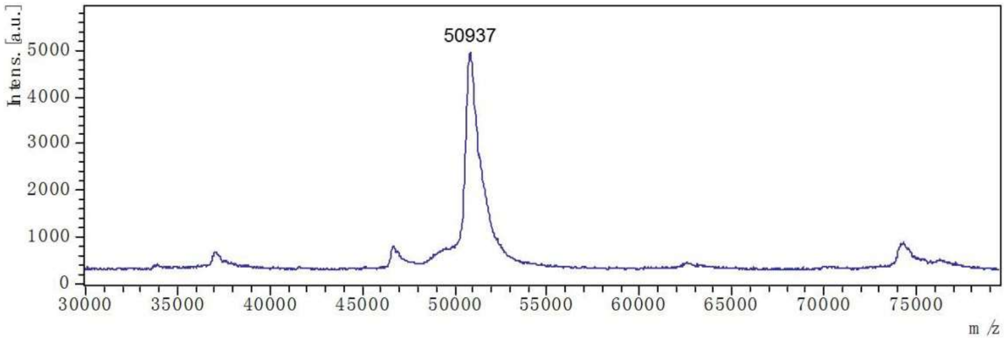

C

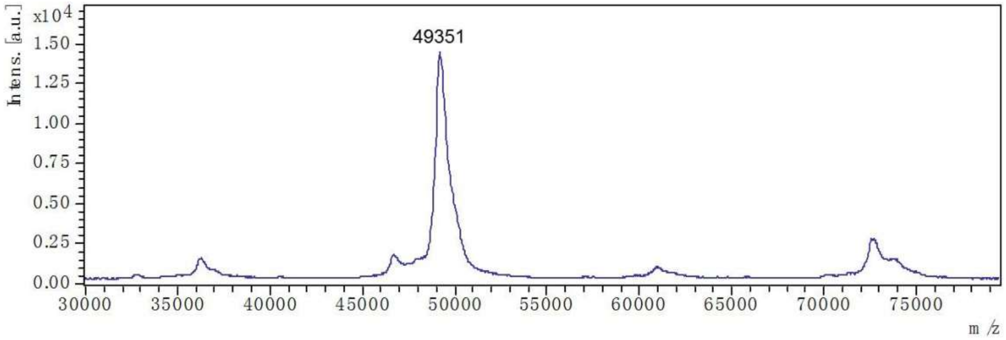

Supplementary Figure 8. MALDI-TOF-MS analysis of the heavy chain of Herceptin glycovariants with DTT treatment. (A) S0G2-Herceptin. (B) S0G2F-Herceptin generated by the AlfC$_{N243A}$ mutant. (C) GlcNAc-Herceptin generated by EndoS2 and AlfC wild-type enzymes. The MALDI-TOF-MS analysis showed almost complete conversion for the enzymatic reactions; yields indicated isolated yields after a single protein A affinity chromatographic purification and quantification by nanodrop analysis.

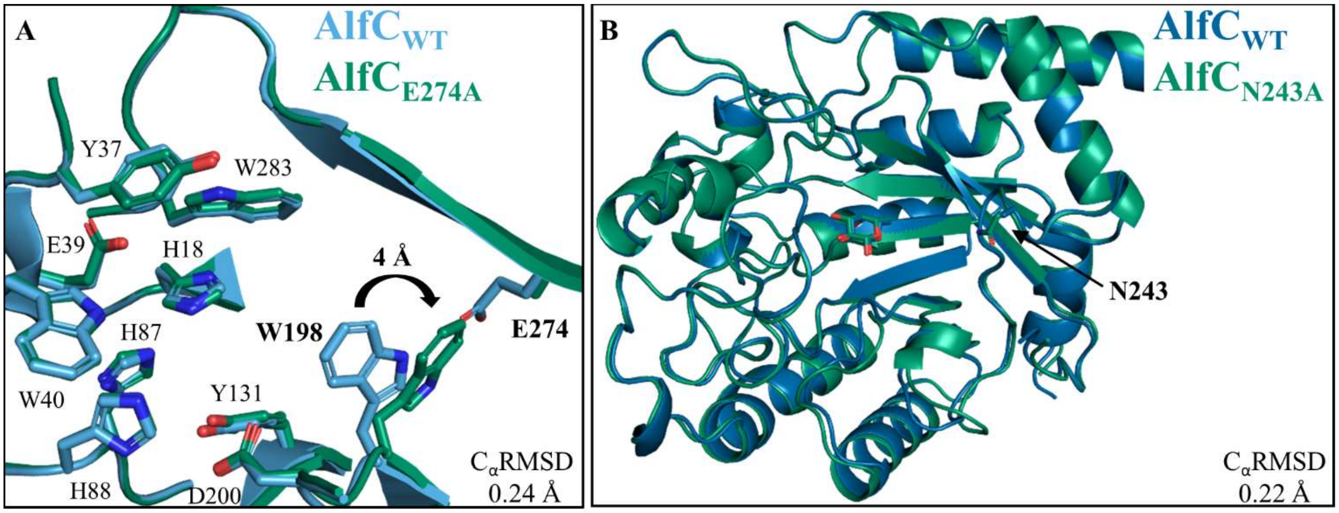

Supplementary Figure 9. Structure of (A) AlfC$_{E274A}$ (green) and (B) AlfC$_{N243A}$ (green) compared to AlfC$_{WT}$ (blue).

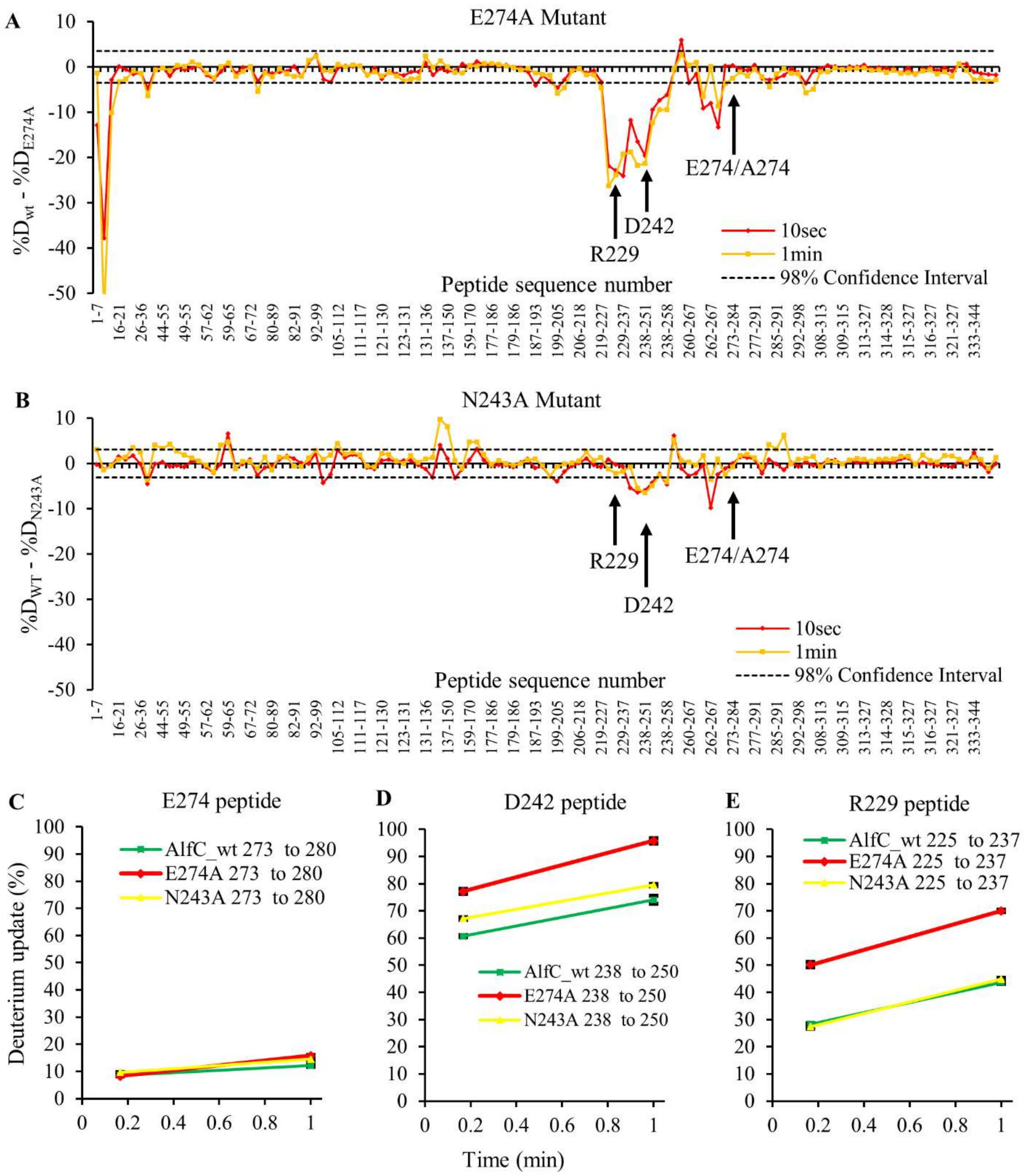

Supplementary Figure 10. HDX-MS of wild type and mutant AlfC E27A and N243A display the solution structure and dynamics of critical motifs of AlfC. **(A-B)** Deuterium uptake difference plots were plotted for wild-type AlfC vs E274A **(A)** and wild type AlfC vs N243A **(B)**. Individual peptides were plotted on the x-axis, going from N- to C-terminus based on the starting residue of each peptides. The difference in percent deuteration between the wild-type and mutant is plotted for each peptide following 10 sec (red) and 1 min (yellow) of incubation in deuterated buffer. 98% confidence interval is displayed as dashed lines. Statistically significant differences are mapped on Figure 6C-D. **(C-E)** Percent deuterium uptake traces for representative peptides from critical motifs including residue 274 **(C)**, residues 242 and 243 **(D)**, and residue 229 **(E)** in the context of wild-type AlfC (green), E274A mutant (red) and N243A mutant (yellow). Percent standard deviation are shown in black error bars.

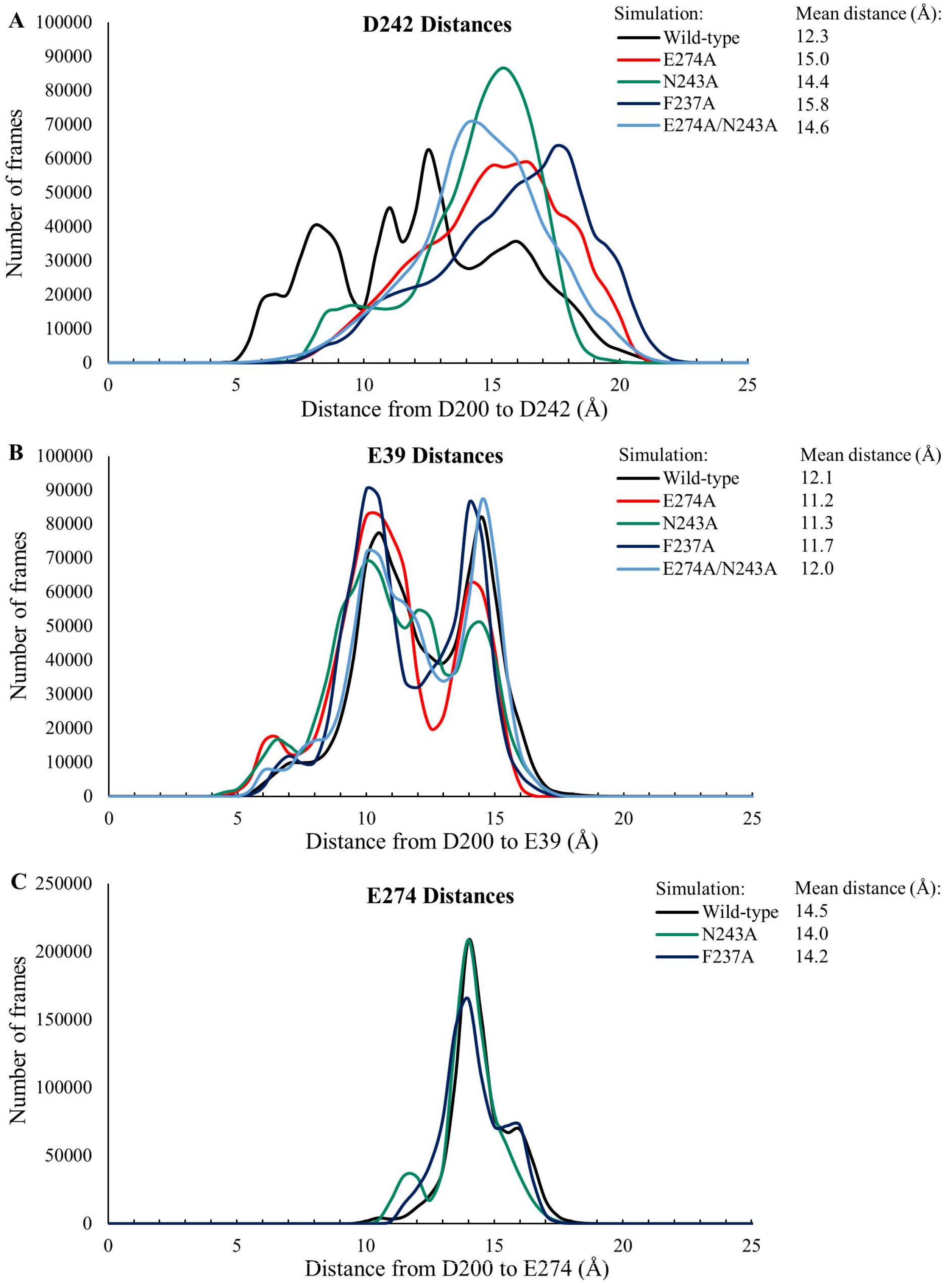

Supplementary Figure 11. Molecular dynamic simulation of AlfC transfucosylation mutants. Frames are plotted by distance separating O_δ of D200 and **(A)** O_δ of D242; **(B)** O_γ of E39; **(C)** O_γ of E274.

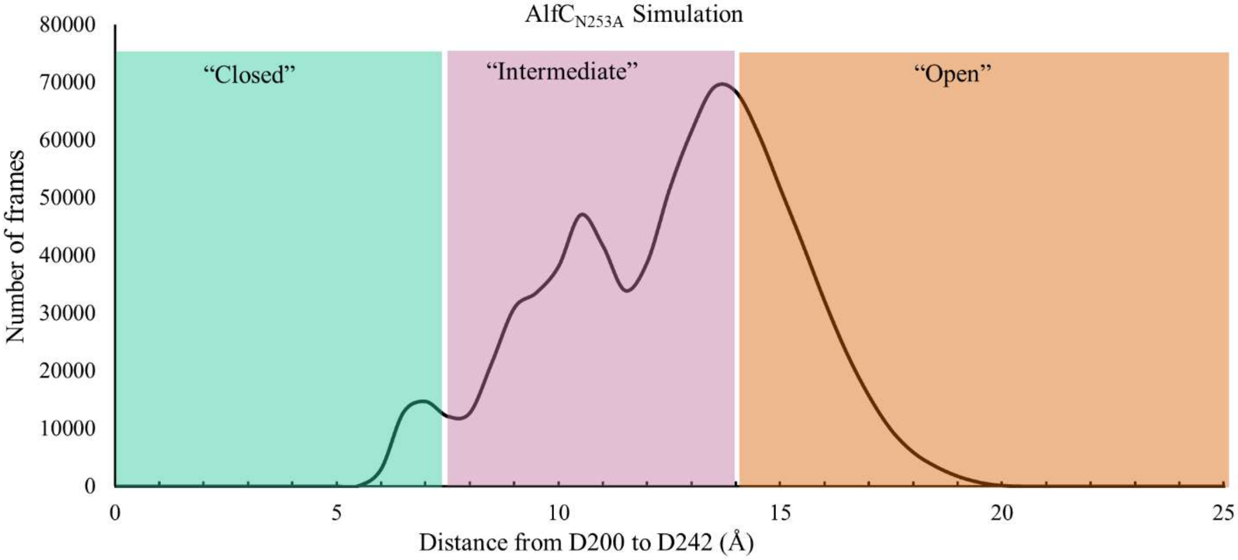

Supplementary Figure 12. Molecular dynamic simulation of AlfC$_{N253A}$ with frames plotted by distance separating AlfC D200 and D242 O_{δ}.

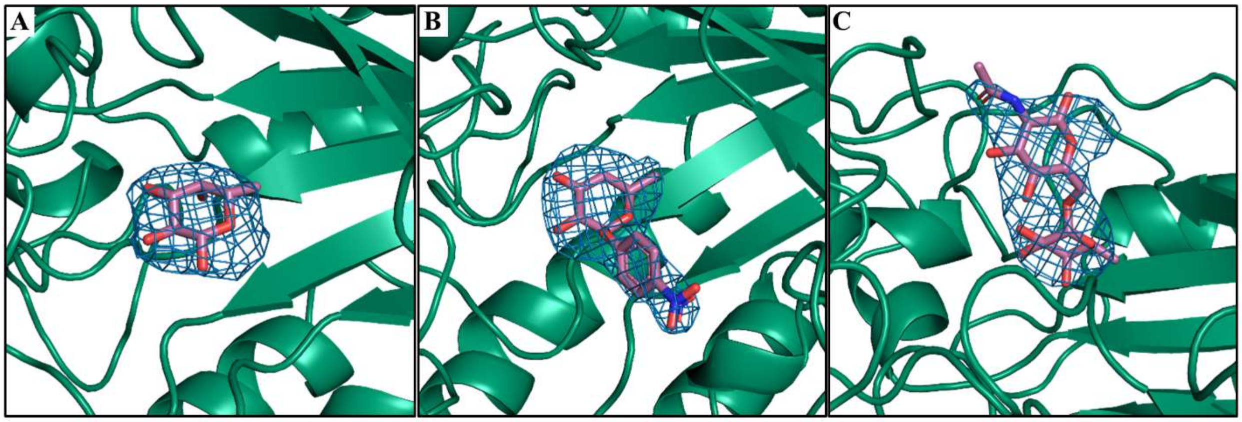

Supplementary Figure 13. Polder maps were generated by omitting ligand atoms. **(A)** Fucose, σ = 5.5, carve = 2.3 Å; **(B)** 4NP-fuc, σ = 4, carve = 2.3 Å; **(C)** Fucα(1,6)GlcNAc, σ = 4, carve = 2.6 Å

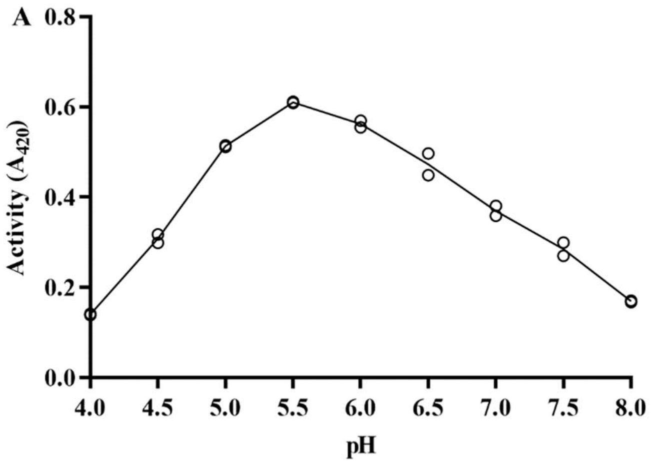

Supplementary Figure 14. AlfC pH dependence on 4NP-fuc hydrolysis using 100 mM PCB buffer system (sodium propionate, sodium cacodyolate, bis-tris propane). Separate reactions were measured in duplicate.

Enzyme	Subfamily	Nucleophile	Acid/base candidate 1	Acid/base candidate 2	Acid/base candidate 3	PDB code	Reference
Crystal structure available							
BT2192	B	D188	E234#	-	E275	3eyp	N/A
BT3798	B	D199	E240#	-	-	3gza	N/A
BiAfcB	B	D172	E217#	E46	E281	3ues	[7]
BACOVA_04357	B	D204	E249#	E78	E305	4zrx	N/A
GH29_0940	B	D162	E220#	E37	E257	5k9h	[26]
FgFCO1	A	D226	E288#	E46	-	4ni3	[23]
α-L-f1wt	A	D186	E239#	E34	E258	6gn6	[18]
Crystal structure and mutagenesis/kinetics available							
Tmα-fuc	A	D224	E266#*	E66	E281	1hl8	[20]
BT2970	A	D229	E288#*	E87	E306	2wvv	[8]
AlfC	A	D200	D242	E39	E274	6o18	This work
Mutagenesis/kinetics available							
Ssα-fuc	A	D242	E292?	E58*	E292?	N/A	[9]
FucA1	A	D225	-	E70?	E289*	N/A	[10]
NcFuc	A	D214	-	E59*	E278	N/A	[6]
BT4136	B	D204	E249*	E78?	E305	N/A	[5]
BT1625	B	D204	E249*	E78?	E305	N/A	[5]
cFase I	B	D242	E302	E68?	E315*	N/A	[25]

Supplementary Table 1. GH29 α-fucosidases which have been subjected to acid/base analysis through structure and/or mutagenesis. Acid/base candidate 1 refers to the residue that aligns by sequence or structure with the acid/base of Tmα-fuc; Candidate 2 aligns with the acid/base of Ssα-fuc; Candidate 3 aligns with the acid/base of FucA1. The most likely acid/base residue has been bolded. #, structural evidence; *, chemical evidence (azide rescue).

Data Collection	Unliganded $AlfC_{WT}$	$AlfC_{WT}$ -Fucose	$AlfC_{D200A}$-4NP-Fuc
Resolution range	29.09 - 2.55 (2.64 - 2.55)	29.82 - 2.6 (2.69 - 2.6)	39.43 - 2.6 (2.69 - 2.6)
Space group	$C222_1$	$C222_1$	$P2_12_12_1$
Unit cell (a, b, c, α, β, γ)	89.30, 137.12, 264.71, 90, 90, 90	89.98, 138.24, 264.44, 90, 90, 90	89.73, 139.08, 263.37, 90, 90, 90
Total reflections	291045 (29172)	245543 (25604)	490615 (47731)
Unique reflections	53116 (5271)	50858 (5036)	99099 (9887)
Multiplicity	5.5 (5.5)	4.8 (5.1)	5.0 (4.8)
Completeness (%)	100 (100)	99.0 (100)	96.0 (99.0)
Mean I/sigma(I)	14.53 (1.07)	15.29 (1.12)	8.46 (0.93)
Wilson B-factor	80.60	87.39	65.77
R_{merge}	0.059 (1.26)	0.054 (1.29)	0.115 (1.45)
R_{meas}	0.066 (1.39)	0.061 (1.43)	0.129 (1.63)
CC1/2	0.999 (0.57)	0.999 (0.495)	0.995 (0.651)
CC*	1 (0.85)	1 (0.814)	0.999 (0.888)
Refinement			
Reflections used in refinement	53110 (5271)	50853 (5034)	97923 (9831)
Reflections used for R-free	2621 (263)	2486 (236)	4987 (514)
R_{work}	0.208 (0.406)	0.209 (0.350)	0.2315 (0.398)
R_{free}	0.242 (0.400)	0.228 (0.388)	0.2607 (0.411)
CC(work)	0.951 (0.465)	0.960 (0.596)	0.937 (0.724)
CC(free)	0.964 (0.421)	0.955 (0.476)	0.928 (0.686)
Number of non-hydrogen atoms	10225	10255	20999
Macromolecules	10192	10198	20607
Ligands	0	44	160
Real space CC ligands	N/A	0.94	0.89
Protein residues	1300	1291	2584
Waters	33	13	232
RMS(bonds)	0.014	0.013	0.014
RMS(angles)	1.73	1.65	1.72
Ramachandran favored (%)	93	95	94
Ramachandran allowed (%)	6.5	4.9	5.9
Ramachandran outliers (%)	0.4	0.55	0
Rotamer outliers (%)	3.6	5.6	3.2
Average B-factor for macromolecules	101.98	113.43	77.98
Average B-factor for ligands	N/A	102.09	77.81
PDB Code	6O18	6O1A	6O1C

Supplementary Table 2. Data collection and refinement statistics. Overall values are reported with highest resolution shell in parentheses.

Data Collection	$AlfC_{E274A}$	$AlfC_{N243A}$-Fucose	$AlfC_{D200A}$-Fuc-α(1,6)GlcNAc
Resolution range	29.12 - 3.55 (3.68 - 3.55)	47.75 - 2.0 (2.071 - 2.0)	29.04 - 3.14 (3.252 - 3.14)
Space group	$C222_1$	$P2_1$	$C222_1$
Unit cell (a, b, c, α, β, γ)	87.75, 139.3, 265.59, 90, 90, 90	80.30, 259.92, 80.35, 90, 118.81, 90	89.02, 139.25, 263.38, 90, 90, 90
Total reflections	89921 (9164)	481076 (25229)	143262 (13548)
Unique reflections	18262 (1827)	179997 (11710)	28911 (2824)
Multiplicity	4.9 (5.0)	2.7 (2.0)	5.0 (4.8)
Completeness (%)	91 (93)	91.4 (60.5)	1.00 (1.00)
Mean I/sigma(I)	9.63 (0.81)	9.96 (0.71)	6.59 (0.91)
Wilson B-factor	123.68	39.92	88.81
R_{merge}	0.1472 (2.635)	0.065 (0.89)	0.1579 (1.554)
R_{meas}	0.1636 (2.921)	0.080 (1.13)	0.1764 (1.746)
CC1/2	0.997 (0.314)	0.997 (0.515)	0.995 (0.439)
CC*	0.999 (0.691)	0.999 (0.824)	0.999 (0.781)
Refinement			
Reflections used in refinement	18262 (1822)	176953 (11664)	28911 (2822)
Reflections used for R-free	913 (91)	9035 (574)	1445 (153)
R_{work}	0.2463 (0.3737)	0.2301 (0.4080)	0.2785 (0.4114)
R_{free}	0.2614 (0.3985)	0.2426 (0.4248)	0.2986 (0.3821)
CC(work)	0.953 (0.574)	0.877 (0.575)	0.954 (0.481)
CC(free)	0.938 (0.338)	0.866 (0.437)	0.958 (0.580)
Number of non-hydrogen atoms	10197	21640	10367
Macromolecules	10197	20791	10267
Ligands	0	88	100
Real space CC ligands	N/A	0.88	0.89
Protein residues	1296	2590	1289
Waters	0	761	0
RMS(bonds)	0.013	0.014	0.007
RMS(angles)	1.59	1.78	1.26
Ramachandran favored (%)	94.1	95.4	93.5
Ramachandran allowed (%)	5.9	4.3	5.7
Ramachandran outliers (%)	0	0.3	0.8
Rotamer outliers (%)	2.4	3.5	1.4
Average B-factor for macromolecules	105.35	53.09	126.62
Average B-factor for ligands	N/A	55.73	109.53
PDB Code	6O1I	6O1J	6OHE

Supplementary Table 2. Data collection and refinement statistics. Overall values are reported with highest resolution shell in parentheses.
