# Supplementary material for: Structure and dynamics of an α-fucosidase reveal a mechanism for highly efficient IgG transfucosylation
Source: Nat Commun. 2020 Dec 4;11:6204. doi: 10.1038/s41467-020-20044-z (PMC7718225; doi:10.1038/s41467-020-20044-z)
Supplement: Supplementary file 2 — Peer Review File [file 41467_2020_20044_MOESM2_ESM.pdf]

REVIEWER COMMENTS</B>

Reviewer #1 (Remarks to the Author):

The manuscript titled “Structure and dynamics of AlfC reveal mechanism for highly efficient IgG transfucosylation” focuses on description of molecular mechanism of AlfC fucosidase and AlfC mutants transfucosidase activity, which is potentially very interesting for modification of complex glycans occupying human immunoglobulins. The output of the study should lead to general aspect of transfucosidase designing based on fucosidase mutants for creation of α1-6 linkage and possibly other type of fucose linkage to nascent glycan.
The study follows the previously published data by Li C. et al (JACS 2017) showing single point α1,6-fucosidase mutant E274A, which effectively transfers fucose to intact N-glycans. The authors prepared several AlfC mutants to shed lights on the mechanism of hydrolytic and transfucosidase activity of the enzymes. They solved the X-ray crystal structure of wild type AlfC. The mutant N243A revealed almost the same transfucosidase activity as the previously described E274A mutant, other mutants were less active. The authors demonstrate that conformation change of one loop from “closed” to “open” is responsible for conversion from hydrolytic to transfucosidase activity.
I find the results of study interesting supported by many experimental data. However, I do not think that the data show a generalizable mechanism for creation of transfucosidases from AlfC that could be used for synthesis of new transfucosidases with different linkage specificity. The authors mentioned in the last sentence of the Results that just shifting the enzyme dynamics towards “open” conformations must be taken with a care. There were no straightforward rules postulated how to design specific transfucosidases from AlfC. A paper published by Zeuner B. et al. “Loop engineering of an α-1,3/4-L-fucosidase for improved synthesis of human milk oligosaccharides” also demonstrates conformational changes of a loop in mutated GH29 fucosidases. I miss in the presented manuscript a novelty that will sheds light on the strategy of new transfucosidases preparation. For this reasons I suggest to not publish this study in Nature Communications. I think that the data will better fit in other peer-reviewed scientific journal such as Structure.

Reviewer #2 (Remarks to the Author):

The manuscript describes an MS and structural study of the Lactobacillus casei GH29A α-fucosidase, AlfC, to understand its hydrolysis mechanism and the transfucosylation activity of specific mutants. In my opinion the structural (crystallographic) data are quite informative in terms of characterization of the AlfC and its potentials for opening and closing. However, I find the MS data in Figure 1, which appear at the very start of the paper and are presumably central to it, quite problematic. Also, I find that the MD are very limited in terms of sampling, therefore any mechanistic conclusions drawn from such short and unbiased simulations quite simplistic. Finally, I believe the paper could also benefit from the acknowledgement that alpha fucosidases have actually very different 3D folds. A few more details below.

The authors discuss initially the division of the GH29 enzymes family in two subfamilies, based on the substrate specificity. I think that is also quite important to underline that GH29 families differ fundamentally in their 3D structure (or fold). For example, the human alpha-fucosidase as well as BKF have a typically eukaryotic fold (see Sulzenbacher et al, (2004) J Biol Chem 279: 131190, PDB 1odu) and a broad specificity, with a kinetic preference for 1-6/2. Bacterial gh29 such as BT2970 and the AlfC have a completely different fold and very little (non-significant) sequence identity. So it is very likely that these two types of enzymes follow different mechanisms. Also, the necessity to remove the N-glycan’s antennae with EndoS/S2 before fucose hydrolysis with an alpha fucosidases, is very likely linked to the enzyme fold and its specific steric constraints, see point on line 84 page 4. For example, eukaryotic fucosidases (with PDB 1ODU fold) have a really narrow and pokey binding site that is seriously problematic in case of bulky substrates (see O’Flaherty et al, J. Proteome Res (2017)

16:4237).

The activity of AlfC in hydrolysing fucose from the Fc glycan of Rituximab has been monitored by MS. The results are shown in Figure 1C and 1D with what seems to me a “drawn” graph, which I am not sure what it should represent, but that is definitely not an MS spectrum and should not be labelled as such. Further down in the Method section details of how the authors actually did the MS of the intact antibody are completely lacking. The structural (crystallographic) data seems complete and well presented to me and the MD simulations, although far from conformational sampling with only 100 ns trajectories, are an interesting complement to support the stability of the structure. Note, in regards to salt bridges observed in MD (page 9 lines 224-225) it’s important to underline that empirical force fields tend to overestimate the strength of salt-bridge interactions (see Ahamed et al PeerJ. 2018; 6: e4967).

Minor points

Page 1, Line 23 The a(1-6) fucose is not exclusive to human or mammalian N-glycans, see for example Paschinger and Wilson, Parasitology (2019) for an exhaustive review.

Page 2, Lines 46 to 49. the authors should underline that fucosidases from class A and B may also have different folds and sequences, i.e. virtually no sequence identity. In fact, GH29-A are common in eukaryotes and GH29-B are generally of bacterial origin. Notably, a broad specificity class B fucosidase from O. bacterium has been identified and characterized relatively recently, more details here Vainauskas et al, Scientific Reports 2018, 8.

Reviewer #3 (Remarks to the Author):

Core-fucosylation play a significant role in glycobiology and in production of therapeutic antibodies. The manuscript from Wang, Sundberg and co-workers describe a solid study exploring the mechanism of the core fucose fucosidase AlfC and their mutants. The study also explore AlfC mutants that may act as a transfucosidase and additionally tolerate complex type N-glycans in contrast to the WT AlfC fucosidase. The authors investigate the mechanisms behind why the diffrent mutants can have different functions and substrate scope by deteremination of crystal structures, MD calculations, kinetic analysis, NMR and HDX-MS analysis. I would recommend publication after minor revision.

Figure S9: color assignment of WT vs mutant not the same in figure as in figure legend, please correct.

The authors make co-crystal structures with the Fuc-a-1,6-GlcNAc disaccharide, it would be desirable to use the disaccharide attached to a peptide backbone for ligand co-crystalization to confirm that the ligand is correctly oriented with regard to steric clash and conclusions explaining why other Fuc linkage connections would not be suitable.

With regard to AlfC mutants acting as transglycosidases, it would have been convincing to additionaly include a core fucose complex type N-glycan as ligand in a co-crystal structure that would confirm that the oligosaccharide fit in the more open conformation of the enzyme.

Regarding the transglycosidase activity, have other complex type N-glycans than biantennary been used as substrates for the fucose translycosylation?

The manuscript is rather long.

Reviewer #4 (Remarks to the Author):

This paper provides a careful and characterization of the structural enzymology of an enzyme that is important for its role in cell adhesion and blockage thereof and post-translational modifications of potential bioactive molecules. The authors provide a credible explanation for the relative rates of hydrolysis versus transglycosylation and made mutant versions that further probe the mechanism of action and support the rationales provided. The MD and HD exchange data buttress the arguments about the role of more open or closed states. Altogether the paper represents an impressive array of experiments that are well integrated into a compelling story.

Technical points to be addressed before any publication:

For solid validation of the bound carbohydrates:
1)The crystallographic "Table 1" must list the real space CC's or Z scores for the bound carbohydrate ligands.
2)Supplemental information should contain polder maps of the bound ligands.
3)The PDB entry ID's must be provided for the newly deposited works.

George N. Phillips, Jr.

We would like to thank the reviewers for their thorough and helpful critiques of our work. Below, we provide point-by-point responses to their comments.

Reviewer 1:

The manuscript titled "Structure and dynamics of AlfC reveal mechanism for highly efficient IgG transfucosylation" focuses on description of molecular mechanism of AlfC fucosidase and AlfC mutants transfucosidase activity, which is potentially very interesting for modification of complex glycans occupying human immunoglobulins. The output of the study should lead to general aspect of transfucosidase designing based on fucosidase mutants for creation of α1-6 linkage and possibly other type of fucose linkage to nascent glycan.

The study follows the previously published data by Li C. et al (JACS 2017) showing single point α1,6-fucosidase mutant E274A, which effectively transfers fucose to intact N-glycans. The authors prepared several AlfC mutants to shed lights on the mechanism of hydrolytic and transfucosidase activity of the enzymes. They solved the X-ray crystal structure of wild type AlfC. The mutant N243A revealed almost the same transfucosidase activity as the previously described E274A mutant, other mutants were less active. The authors demonstrate that conformation change of one loop from "closed" to "open" is responsible for conversion from hydrolytic to transfucosidase activity.

I find the results of study interesting supported by many experimental data. However, I do not think that the data show a generalizable mechanism for creation of transfucosidases from AlfC that could be used for synthesis of new transfucosidases with different linkage specificity.

Thank you for your interest in our work. Whether or not we've discovered a mechanism that is generalizable to other alpha-fucosidases is an open question that we are actively pursuing in our lab. We have been careful to suggest, for now, only that it *may* be generalizable. In the pursuit of novel transfucosidases, no approach has yet proven universally generalizable. The fucosynthase approach (mutating the nucleophile) was unsuccessful with AlfC[1]. In hindsight, the fucoligase approach (mutating the acid/base) may have been fortuitous[1] in light of the new data presented within this paper suggesting E274 may not be the acid/base. In contrast, shifting the dynamics of AlfC was successful and generalizable within this system.

We believe that there is sufficient evidence to suggest that this approach *may* be generalizable to other alpha-fucosidases. Specifically, Tmα-fuc (an enzyme with different linkage specificity), can be converted to a transfucosidase though a set of mutations (T264A, Y267F, L322P)[2] that are structurally similar to the transfucosidase mutants discovered using our approach. While not conclusive, we find these similarities provocative and worthy of further investigation in other enzymes. Where fucosynthase and fucoligase approaches fail, the dynamic approach may be successful.

The authors mentioned in the last sentence of the Results that just shifting the enzyme dynamics towards “open” conformations must be taken with a care. There were no straightforward rules postulated how to design specific transfucosidases from AlfC.

While not discussed explicitly, we believe that the general strategy can be inferred from our approach with the transfucosidase mutant F237A, and from the discussion of similarities with Tmα-fuc:

- **When crystal structures exist, try mutating residues that form contacts between the beta barrel and acid/base loop. Also, try mutating the residue that follows the acid/base residue.**
- **When crystal structures do not exist, try mutating residues that align by sequence and/or correspond by homology modeling with the above residues from other alpha fucosidases that do have structures (e.g., the equivalents of AlfC F237, N243, E274 or Tmα-Fuc T264, Y267, L322).**
- **Take care by testing mutations individually before combining mutations.**

Because of the length of the paper, we have opted not to expand on this any further, but it may be the subject of future publications on the generalizability of the approach.

A paper published by Zeuner B. et al. “Loop engineering of an α-1,3/4-L-fucosidase for improved synthesis of human milk oligosaccharides” also demonstrates conformational changes of a loop in mutated GH29 fucosidases.

The paper referenced by this reviewer is an interesting paper in which the authors made one GH29B fucosidase (*Bb*AfcB) as efficient at transfucosylation as another (*Cp*Afc2) by substituting a loop from the latter into the former[3]. The reviewer might find it interesting that the loop they substituted is the continuation of the acid/base loop. The authors in the referenced paper provide no data to explain why this works, but speculate that it is due to enhanced hydrolytic shielding of the active site. We have shown that the dynamics of the acid/base loop are sensitive to mutation and modulate the transfucosylation efficiency of the enzyme. It may be that seemingly disparate approaches share a common underlying mechanism, elucidated for the first time in our manuscript. If true, this would speak further to the generalizability of this strategy. Accordingly, we have now mentioned and referenced the Zeuner *et al.* paper in the revised manuscript.

I miss in the presented manuscript a novelty that will sheds light on the strategy of new transfucosidases preparation. For this reasons I suggest to not publish this study in Nature Communications. I think that the data will better fit in other peer-reviewed scientific journal such as Structure.

The novelty of our manuscript lies only in part in the mechanism by which novel transfucosidase mutants work. Aside from this mechanism, other important novelties of this manuscript include:

- **The first structure of an α(1,6)-fucosidase**
- **The first crystallographic study to provide rationale for the specificity of a GH29A enzyme.**

- **The first mechanistic study of an antibody-modifying fucosidase**
- **The first detailed dynamic study of the acid/base loop in an α-fucosidase**

Reviewer 2:

The manuscript describes an MS and structural study of the Lactobacillus casei GH29A α-fucosidase, AlfC, to understand its hydrolysis mechanism and the transfucosylation activity of specific mutants. In my opinion the structural (crystallographic) data are quite informative in terms of characterization of the AlfC and its potentials for opening and closing. However, I find the MS data in Figure 1, which appear at the very start of the paper and are presumably central to it, quite problematic. Also, I find that the MD are very limited in terms of sampling, therefore any mechanistic conclusions drawn from such short and unbiased simulations quite simplistic. Finally, I believe the paper could also benefit from the acknowledgement that alpha fucosidases have actually very different 3D folds. A few more details below.

We are glad you found the data informative, and we thank you for your careful read of our manuscript. Detailed responses follow.

The authors discuss initially the division of the GH29 enzymes family in two subfamilies, based on the substrate specificity. I think that is also quite important to underline that GH29 families differ fundamentally in their 3D structure (or fold).

Since the terms "3D structure" and "fold" can be interpreted in at several ways, we are unsure precisely what this reviewer is saying. There are several possibilities:

1) **Referring to the secondary and tertiary structure. For example, AlfC has a $(\beta/\alpha)_8$ fold that is present in the crystal structure of every alpha fucosidase solved to date. Since this fold is fundamentally conserved, we assume the reviewer is referring to the another definition.**
2) **Referring to quaternary structure (ie., multimeric assembly). The multimeric assembly of alpha fucosidases indeed varies widely. We also felt this important to highlight, and depicted it in Figures 2C-E.**
3) **Referring to active site structure. The active site, including catalytic residues, is generally conserved, but there are important differences in surrounding residues that likely dictate differential substrate specificity of each enzyme. In AlfC, this is illustrated in Figure 5A.**

For example, the human alpha-fucosidase as well as BKF have a typically eukaryotic fold (see Sulzenbacher et al, (2004) J Biol Chem 279: 131190, PDB 1odu) and a broad specificity, with a kinetic preference for 1-6/2.

The reviewer mentions several alpha fucosidases in this sentence:

1) **Human alpha fucosidases (of which, there are two: FucA1 and FucA2)**
2) **Bovine kidney fucosidase (BKF)**
3) **Thermotoga maritima alpha-fucosidase (Tmα-fuc; pdb 1ODU)[4]**

Tmα-Fuc is bacterial in origin, but is the closest bacterial homologue of the eukaryotic fucosidases mentioned above, sharing 38% sequence identity with FucA1[4]. For this reason, it has been used in several instances to create homology models of eukaryotic fucosidases. It creates the typical $(\beta/\alpha)_8$ barrel with a C-terminal beta-sandwich and assembles into a hexamer, as depicted in Figure 2E.

We do not believe it is appropriate to call the $(\beta/\alpha)_8$ barrel with a C-terminal beta-sandwich a "typically eukaryotic fold" as it is created by all alpha-fucosidases, prokaryotic and eukaryotic alike (see Table S1 and PDB 3EYP, 3GZA, 3UES, 4ZRX, 5K9H, 4NI3, 6GN6, 1HL8, 2WVV). Nor do we believe it is appropriate to call the hexameric assembly of Tmα-Fuc "typically eukaryotic", as there has only been one eukaryotic high-resoluiton structure solved to date (FgFCO1; PDB 4NI3) and it has been annotated as a monomer by the depositing authors[5]. To our knowledge, there are no high-resolution structures of FucA1, FucA2, or BFK, so it would be premature to comment on their active site structures at any level of resolution that might distinguish them from other alpha fucosidases.

We also do not believe it is appropriate to generalize about "eukaryotic folds" and their relation to substrate specificity. While GH29B enzymes are only active on α(1-3,4) linkages[6], GH29A enzymes have a wider range of specificities[6]. FucA1 and BKF are broadly active on α(1-2,3,4,6) linkages[7], but BFK has a preference for α(1-2,6) as the reviewer mentions. FucA2, however, is active on α(1-2,3,4) linkages[8]. Tmα-fuc (which the reviewer states has a "eukaryotic fold") is active only on α(1-2,3) linkages[2]. The only true eukaryotic protein with a crystal structure is FgFCO1, which is active only on α(1-2)-linkages[5]. While there are undoubtedly important differences in their active sites that control substrate specificity, these differences are yet to be elucidated. We see no broadly generalizable relation between fucosidase origin, fold, and substrate specificity besides what has already been published and cited in our manuscript[6].

Bacterial gh29 such as BT2970 and the AlfC have a completely different fold and very little (non-significant) sequence identity.

We respectfully disagree with this assessment. Percent identity matrices for BT2970, AlfC, and Tmα-fuc (referred to above) are presented below:

**Sequence identity (%)**	**Tmα-fuc**	**AlfC**	**BT2970**
**Tmα-fuc**	**100.00**	**28.00**	**26.40**
**AlfC**	**28.00**	**100.00**	**31.19**
**BT2970**	**26.40**	**31.19**	**100.00**

Pairwise sequence identity varies from 26.4% to 31.2%, which generally corresponds to a conserved overall structure. Indeed, we know this is borne out because crystal structures for all three enzymes exist. Pairwise root mean square deviation in alpha carbons, measured in PyMOL, is presented below:

**Cα RMSD (Å)**	**Tmα-fuc**	**AlfC**	**BT2970**
**Tmα-fuc (PDB 1ODU, chain A)**	**0**	**1.03 Å**	**0.83 Å**
**AlfC (PDB 6O18, chain**	**1.03 Å**	**0**	**1.06 Å**

A)			
BT2970 (PDB 2WVV, chain A)	0.83 Å	1.06 Å	0

Cα RMSDs of ~1 Å or less demonstrate that bacterial and eukaryotic folds are essentially indistinguishable. To further illustrate this point, we have provided a superposition of AlfC (bacterial; cyan) and Tmα-Fuc ("eukaryotic fold"; dark blue), as shown below.

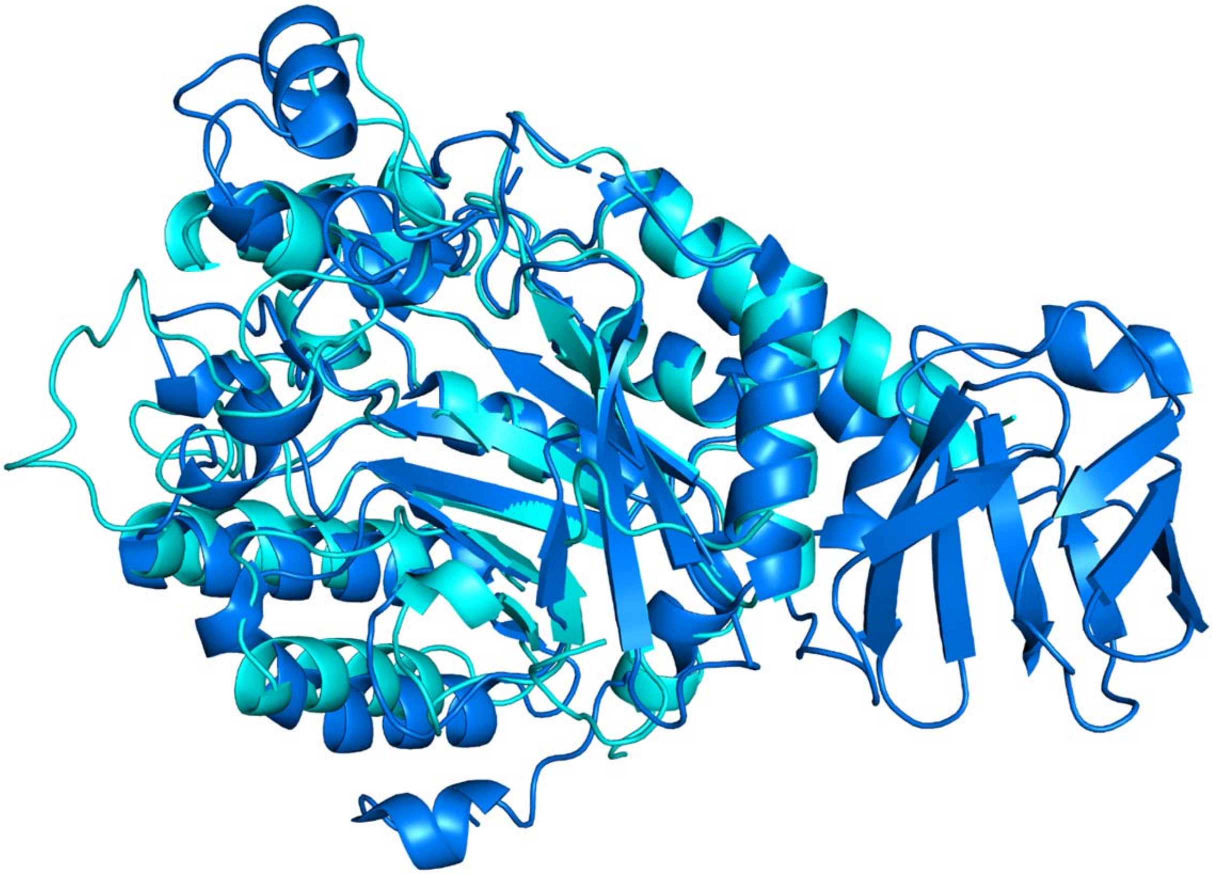

As depicted above, bacterial GH29 such as BT2970 and AlfC have nearly identical overall folds to Tmα-fuc (which the reviewer states as having a typically "eukaryotic fold"). Indeed, the catalytic nucleophile and acid/base residues (yellow) are structurally conserved in all three enzymes (AlfC-cyan, BT2970-magenta, Tmα-fuc-cyan), as shown below

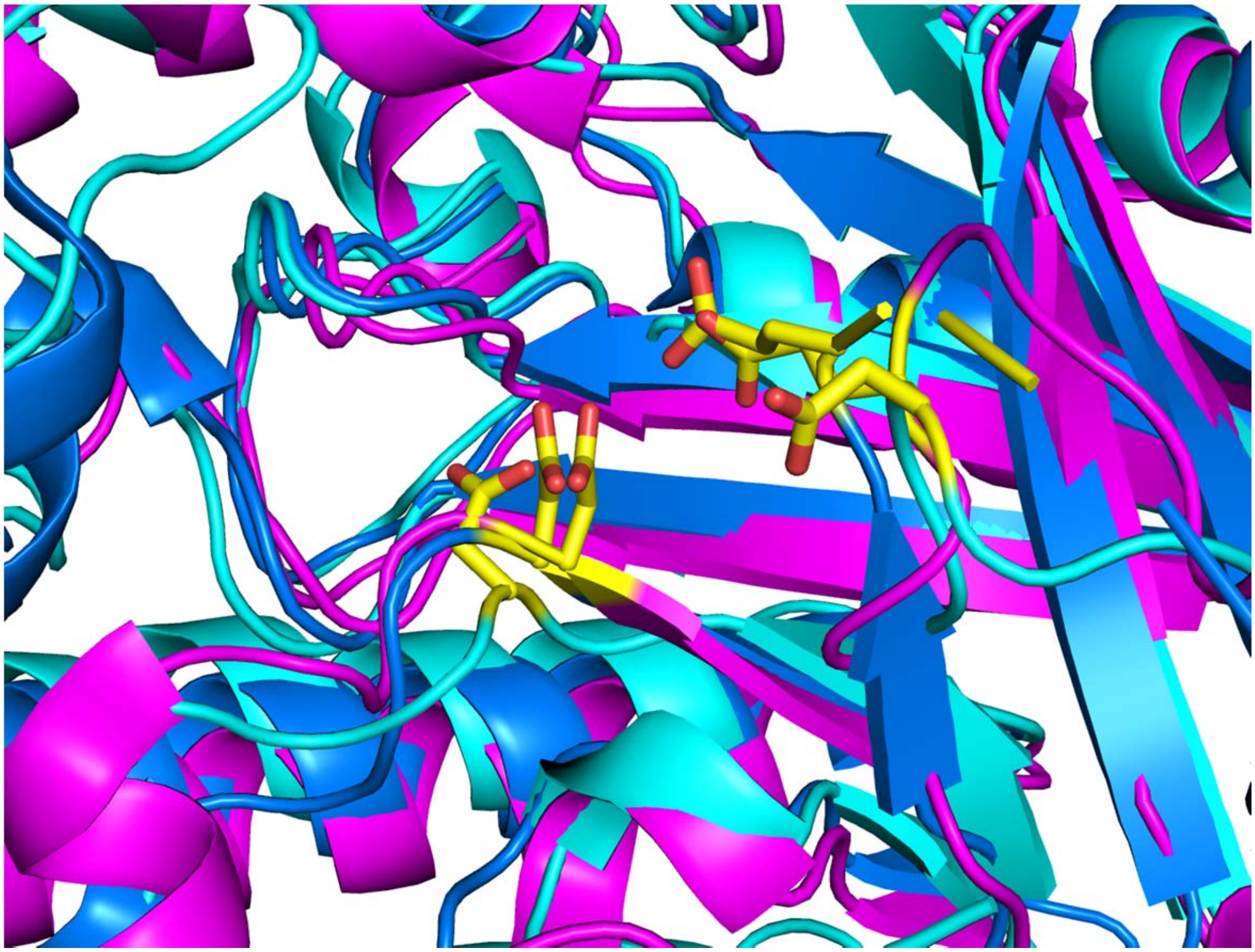

So it is very likely that these two types of enzymes follow different mechanisms.

We respectfully disagree with this assessment. The strong structural conservation of catalytic residues suggests a unified mechanism of hydrolysis. While it remains possible that they employ different mechanisms, it seems unlikely and we believe there is insufficient evidence to suggest so. Perhaps, instead, the reviewer means to suggest that different alpha fucosidases within the same family (GH29A) have different mechanisms of *binding* their substrates. This is almost certainly true as indicated by their different linkage specificities. However, this is likely due to subtle differences in the active site "+1" binding site that recognizes the carbohydrate attached to fucose, which we discuss in Figure 5A.

Also, the necessity to remove the N-glycan's antennae with EndoS/S2 before fucose hydrolysis with an alpha fucosidases, is very likely linked to the enzyme fold and its specific steric constraints, see point on line 84 page 4.

We agree with this point and illustrated this concept in Figure 5A and the accompanying text in the Results section:

“With fucose in the active site, GlcNAc sits in an aromatic subsite adjacent to the active site, formed by residues Y37, W40, and W158. This binding mode furthermore allows oxygen of the N-acetyl group to hydrogen bond with the backbone nitrogen of A154. N-linkages of this disaccharide (as seen on EndoS-treated IgG) can be readily accommodated, as C1 of GlcNAc (which connects to Asn297 of IgG) points away from the surface of the enzyme. In contrast, AlfC cannot accommodate longer carbohydrate linkages (as seen in untreated IgG), because O4 of GlcNAc (which connects to another GlcNAc) would point directly towards the surface of the enzyme, creating steric clashes for any polysaccharide longer than two residues.”

For example, eukaryotic fucosidases (with PDB 1ODU fold) have a really narrow and pokey binding site that is seriously problematic in case of bulky substrates (see O’Flaherty et al, J. Proteome Res (2017) 16:4237).

The eukaryotic fucosidase described in the referenced paper is BKF. In this paper, homology models were created from Tmα-fuc, with which it shares 35% sequence identity[9]. The modeling presented in this paper is reasonable, and their conclusions are plausible. However, in the absence of actual X-ray crystal or other high-resolution structures, we hesitate to comment on the precise shape of an enzyme’s active site, especially since there are flexible and disordered loops in the vicinity that homology models struggle to accurately predict. However, we agree with the general notion that some enzymes have narrower active sites than others, which likely significantly affects the enzyme’s substrate preference.

The activity of AlfC in hydrolysing fucose from the Fc glycan of Rituximab has been monitored by MS. The results are shown in Figure 1C and 1D with what seems to me a “drawn” graph, which I am not sure what it should represent, but that is definitely not an MS spectrum and should not be labelled as such. Further down in the Method section details of how the authors actually did the MS of the intact antibody are completely lacking.

We have published MS data depicted this way previously in numerous peer-reviewed journals, including *Nature Communication* (Figure 9E; left)[10] and *ACS Central Science* (Supplemental Figure 7; right)[11], which we have reproduced below:

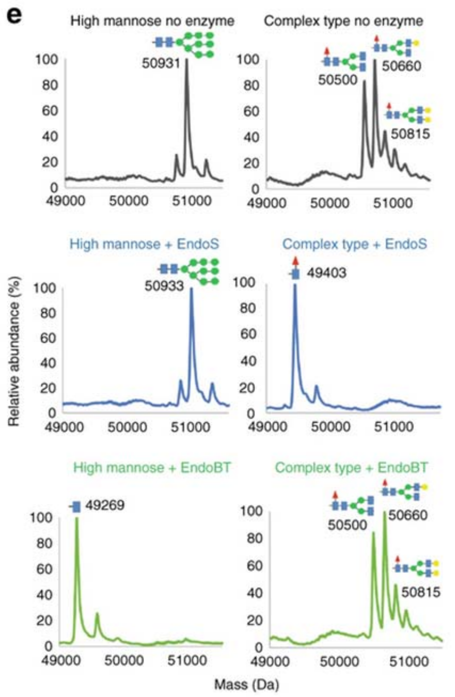

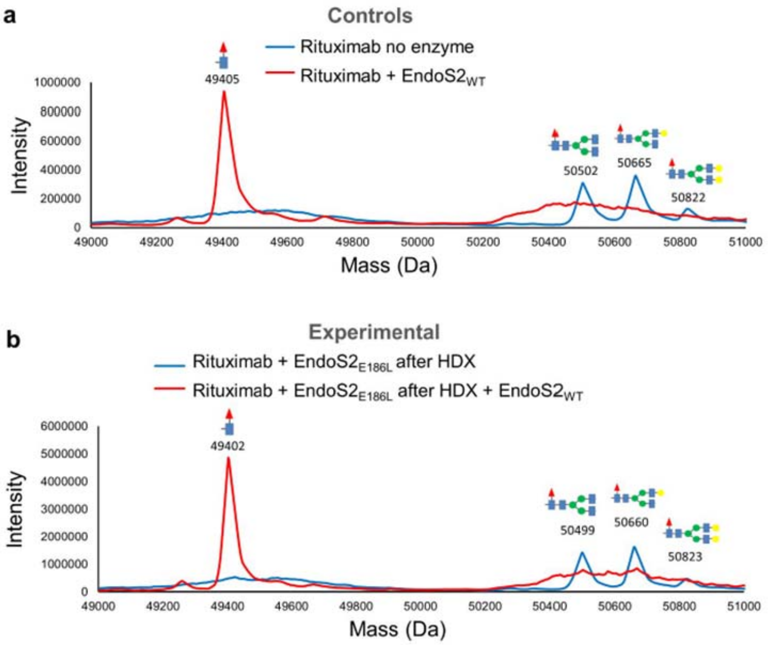

We apologize that, in this instance, we failed to adequately document this process in the Methods section. We have updated this section, which now reads:

"Activity on fully glycosylated Rituximab was measured using 5 µM Rituximab with 1 µM AlfC$_{WT}$ or AlfC$_{E274A}$ in PBS for one month at 25°C. Activity on partially deglycosylated Rituximab was measured using 5 µM Rituximab with 50 nM EndoS2 and 1 µM AlfC$_{WT}$ in PBS overnight at 25°C. EndoS2 rapidly hydrolyzes antibody glycans between the first and second *N*-acetylglucosamine residues, leaving one *N*-acetylglucosamine residue and fucose attached to each heavy chain of the antibody. After the designated incubation, 10 µL aliquots of each reactions were quenched by the addition of 1.1 µL trifluoroacetic acid. The quenched reactions were then mixed with 50 mM TCEP, and analyzed by LC-MS using an Accela LC System attached to a LXQ linear ion trap mass spectrometer (Thermo Scientific, Waltham, MA), as previously described[38]. Relative amounts of substrate and hydrolysis products were quantified after deconvolution of the raw data using BioWorks (Thermo Scientific, Waltham, MA). Relative intensities by mass were then exported to GraphPad Prism and replotted, overlayed, and annotated."

We understand that the figure in this manuscript might appear more "drawn" in quality, perhaps due to the relatively thick lines used. This could also be due to the fact that this spectrum is more zoomed in on the mass range of interest than a typical spectrum, turning the "peaks" we normally associate with MS data into "waves" here. We have decreased the line thickness and zoomed out on the mass range in order to give the figure a less "drawn" quality, reproduced below:

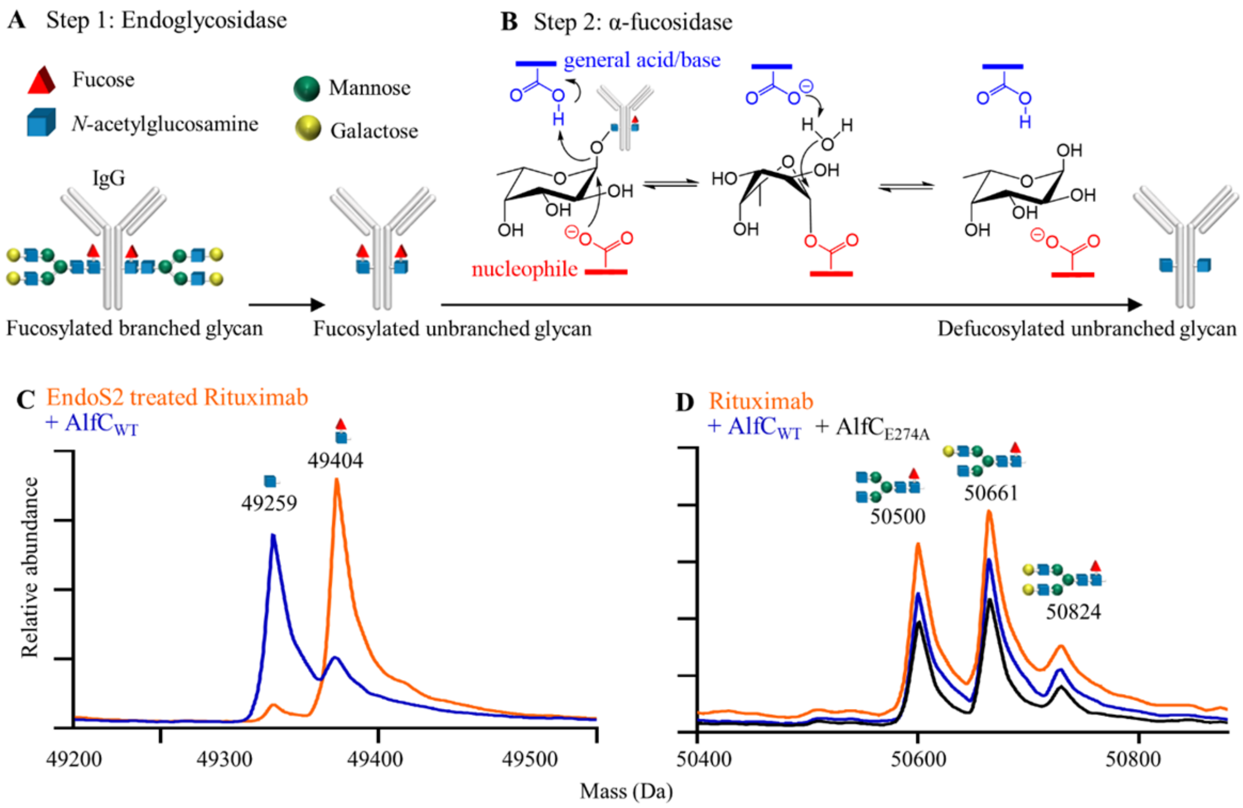

To further demonstrate that this graph represents real MS data, we have provided screenshots of intermediate steps from data acquisition to final graph creation, demonstrating that this format of MS data presentation faithfully represents the raw data while still being easily digestible for a broad audience.

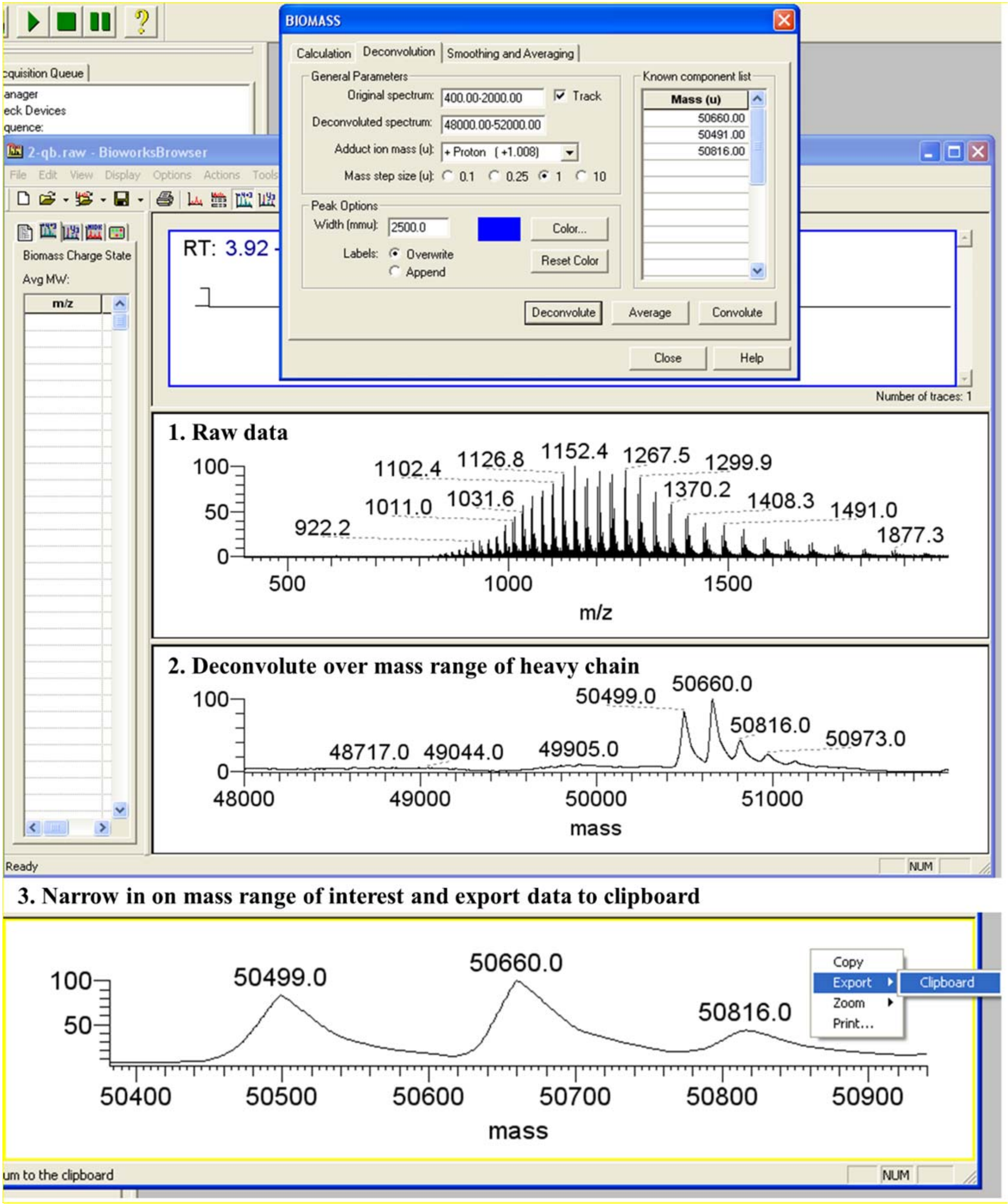
BIOMASS
Calculation
Deconvolution
Smoothing and Averaging
General Parameters
Original spectrum: 400.00-2000.00
Track
Deconvoluted spectrum: 48000.00-52000.00
Adduct ion mass (u): + Proton (+1.008)
Mass step size (u): 0.1 0.25 1 10
Peak Options
Width (mmu): 2500.0
Color...
Labels: Overwrite
Append
Reset Color
Known component list
Mass (u)
50660.00
50491.00
50816.00
Deconvolute
Average
Convolute
Close
Help
2-qb.raw - BioworksBrowser
File Edit View Display Options Actions
Biomass Charge State
Avg MW:
m/z
RT: 3.92
Number of traces: 1
1. Raw data
1102.4 1126.8 1152.4 1267.5 1299.9
1011.0 1031.6 1370.2 1408.3 1491.0
922.2 1877.3
100 50 0
500 1000 1500
m/z
2. Deconvolute over mass range of heavy chain
50499.0 50660.0 50816.0 50973.0
48717.0 49044.0 49905.0
100 0
48000 49000 50000 51000
mass
Ready
NUM
3. Narrow in on mass range of interest and export data to clipboard
50499.0 50660.0 50816.0
100 50
50400 50500 50600 50700 50800 50900
mass
Copy
Export
Clipboard
Zoom
Print...
um to the clipboard
NUM

4. Paste into GraphPad Prism

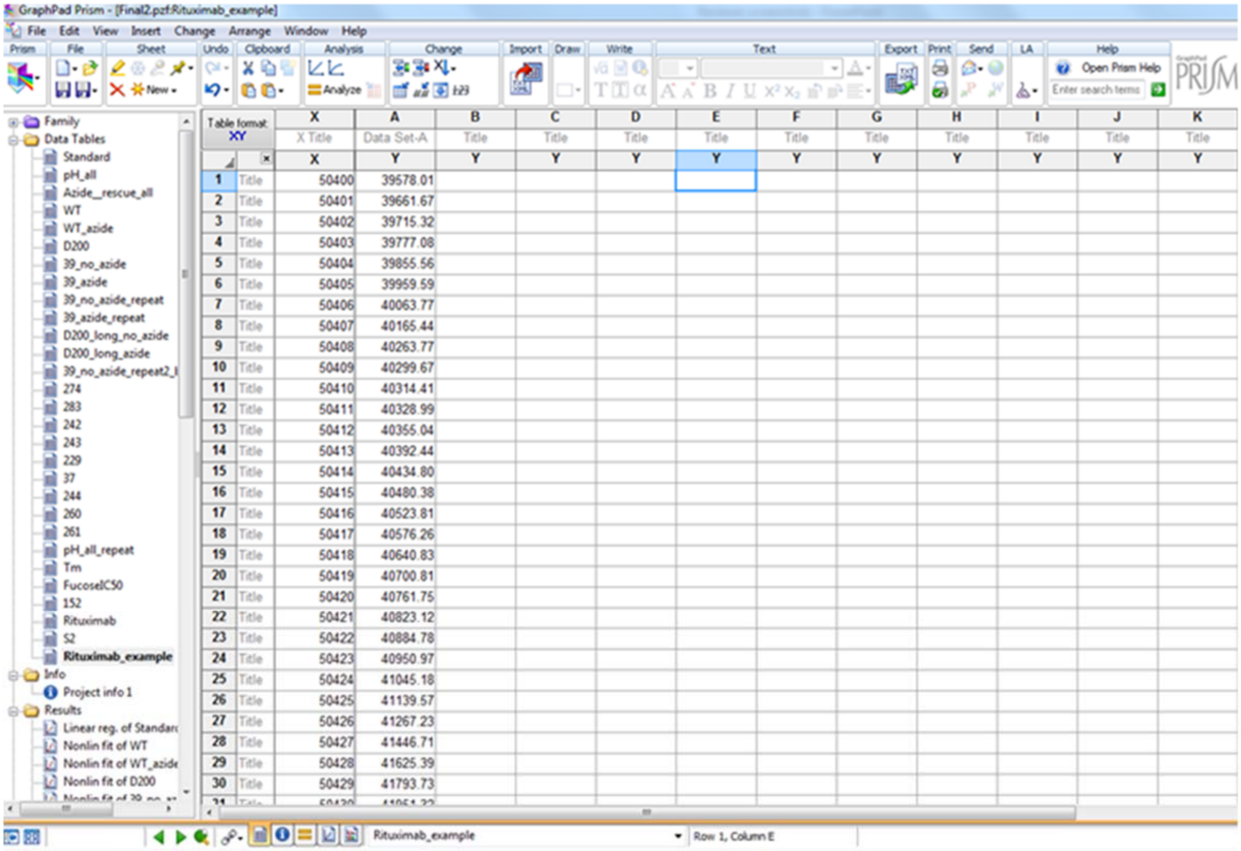

5. Output graph(s) can be overlayed and annotated for final figure

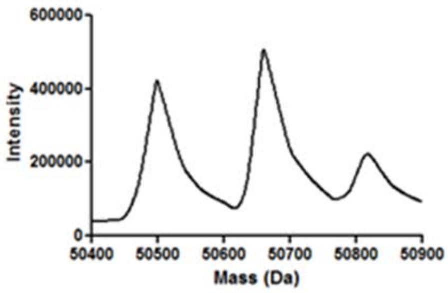

Finally, to ease the concern of this reviewer and any other readers, full source data have been provided for this paper.

The structural (crystallographic) data seems complete and well presented to me and the MD simulations, although far from conformational sampling with only 100 ns trajectories, are an interesting complement to support the stability of the structure.

We appreciate the reviewers concern that 100 ns trajectories are shorter than one might prefer. Unfortunately, these simulations were very computationally expensive, as they had

to be performed on 6 different enzymes (wild-type and 5 point mutants). Additionally, they had to be performed using the full 160kDa AlfC tetramers, as monomers are unstable during simulation. However, because there were four AlfC monomers per 100 ns simulation, there were effectively 400 ns of simulation for each AlfC enzyme. During these simulations, we captured each monomer repetitively cycling between "open" and "closed" conformations, which we felt validated our approach in terms of sampling the conformations relevant to this manuscript. We also ran simulations using enhanced sampling techniques such as augmented MD (aMD) and simulated tempering. We did not notice any major differences using these sampling techniques, so we stuck with classical MD to reduce the potential of distorting the relative abundance of "open" and "closed" conformations.

Note, in regards to salt bridges observed in MD (page 9 lines 224-225) it's important to underline that empirical force fields tend to overestimate the strength of salt-bridge interactions (see Ahamed et al PeerJ. 2018; 6: e4967).

The reviewer mentions a good point that salt-bridge interactions are overestimated by many empirical force fields. However, the referenced paper[12] tested older force fields that were not used in this paper, such as CHARMM22 and CHARMM27. In our modeling, we employed the CHARMM36m force field (now clarified in the methods), which was specifically designed for improved salt-bridge interactions compared to previous force fields[13]. This, combined with strong evidence for the salt-bridge in the form of homologous crystal structures (Figure S2B) gives us increased confidence in this interaction, so we don't feel it necessary to qualify these results.

Minor points

Page 1, Line 23 The a(1-6) fucose is not exclusive to human or mammalian N-glycans, see for example Paschinger and Wilson, Parasitology (2019) for an exhaustive review.

The sentence of concern is, "*Lactobacillus casei* produces an α-fucosidase, AlfC, with specificity towards α(1,6)-fucose, the exclusive linkage in human *N*-glycan core fucosylation."

We did not mean to suggest that core α(1,6)-fucose linkages are *only* found in humans. As the reviewer's reference notes, this linkage is found across plant and animal kingdoms[14].

We also did not mean to suggest that core fucose linkages are always α(1,6) in every species. As the reviewer's reference notes, core α(1,3)-fucose linkages are present in several insects and parasites[14].

However, our sentence, as written, remains true to the best of our knowledge: α(1,6)-fucose is the exclusive linkage found in human *N*-glycan core fucosylation. To prevent future confusion over this phrasing, we have modified the sentence to read as follows:

"*Lactobacillus casei* produces an α-fucosidase, AlfC, with specificity towards α(1,6)-fucose, the only linkage found in human *N*-glycan core fucosylation."

Page 2, Lines 46 to 49. the authors should underline that fucosidases from class A and B may also have different folds and sequences, i.e. virtually no sequence identity.

We feel that this statement is generally misleading. To illustrate this, we have gathered from CAZypedia (http://www.cazy.org/GH29_characterized.html) the sequences of 20 α-fucosidases which have been well-characterized. A percent identity matrix is presented below, with the first 7 α-fucosidases falling into class B, and the subsequent 13 falling into class A.

Percent Identity Matrix - created by Clustal2.1

1: BT3798	100.00	28.02	32.25	31.50	29.25	33.74	33.99	15.87	17.83	16.62	16.62	17.66	17.23	17.71	17.61	19.13	17.63	19.05	21.27	21.91
2: BiAfcB	28.02	100.00	43.85	44.30	44.74	34.92	41.08	17.30	19.68	23.84	17.96	17.98	19.19	17.84	19.84	21.16	18.88	21.28	21.77	24.25
3: BT4136	32.25	43.85	100.00	79.34	79.52	36.94	38.40	13.35	19.13	23.28	20.27	18.73	19.41	18.23	18.85	23.22	22.25	22.91	23.04	25.14
4: BT1625	31.50	44.30	79.34	100.00	85.15	38.82	39.70	16.52	19.67	21.55	19.46	21.76	20.22	19.89	19.40	21.90	21.72	22.55	23.04	25.14
5: BACOVA_04357	29.25	44.74	79.52	85.15	100.00	38.12	39.81	15.61	19.27	21.84	19.61	20.29	19.55	19.21	19.49	22.40	21.45	22.55	22.75	26.21
6: BT2192	33.74	34.92	36.94	38.82	38.12	100.00	41.27	14.97	19.73	19.17	16.03	16.07	17.58	15.98	17.63	21.51	19.43	22.70	19.95	22.44
7: GH29_0940	33.99	41.08	38.40	39.70	39.81	41.27	100.00	16.63	18.60	22.92	15.69	17.82	18.26	18.21	17.66	23.58	23.63	25.90	27.61	27.59
8: FgFCO1	15.87	17.30	13.35	16.52	15.61	14.97	16.63	100.00	32.08	28.78	24.15	24.02	24.63	26.52	28.05	21.55	19.70	18.84	24.47	25.20
9: ssfuc	17.83	19.68	19.13	19.67	19.27	19.73	18.60	32.08	100.00	33.90	31.60	29.69	31.76	30.53	33.81	19.13	21.82	23.79	19.22	21.41
10: TM	16.62	23.84	23.28	21.55	21.84	19.17	22.92	28.78	33.90	100.00	33.73	36.32	35.90	40.14	39.37	25.26	25.99	29.08	29.44	29.69
11: sp|P10901|FUCO_DICDI	16.62	17.96	20.27	19.46	19.61	16.03	15.69	24.15	31.60	33.73	100.00	44.67	43.69	42.30	46.10	21.07	27.23	25.35	27.11	26.86
12: NcFuc	17.66	17.98	18.73	21.76	20.29	16.07	17.82	24.02	29.69	36.32	44.67	100.00	55.61	41.26	46.05	21.14	27.73	27.96	25.07	25.81
13: FUCA1	17.23	19.19	19.41	20.22	19.55	17.58	18.26	24.63	31.76	35.90	43.69	55.61	100.00	42.79	49.43	23.49	29.71	27.24	24.61	24.34
14: tr|I2ERT6|I2ERT6_EMTOG	17.71	17.84	18.23	19.89	19.21	15.98	18.21	26.52	30.53	40.14	42.30	41.26	42.79	100.00	52.38	25.62	26.96	26.15	25.95	27.52
15: KXK31601.1_FUCO	17.61	19.84	18.85	19.40	19.49	17.63	17.66	28.05	33.81	39.37	46.10	46.05	49.43	52.38	100.00	23.15	28.35	27.50	25.60	27.49
16: Elizabethkingia	19.13	21.16	23.22	21.90	22.40	21.51	23.58	21.55	19.13	25.26	21.07	21.14	23.49	25.62	23.15	100.00	24.39	28.90	28.91	28.61
17: BT2970	17.63	18.88	22.25	21.72	21.45	19.43	23.63	19.70	21.82	25.99	27.23	27.73	29.71	26.96	28.35	24.39	100.00	28.21	26.10	28.78
18: AlfC	19.05	21.28	22.91	22.55	22.55	22.70	25.90	18.84	23.79	29.08	25.35	27.96	27.24	26.15	27.50	28.90	28.21	100.00	32.69	32.26
19: Tannerella	21.27	21.77	23.04	23.04	22.75	19.95	27.61	24.47	19.22	29.44	27.11	25.07	24.61	25.95	25.60	28.91	26.10	32.69	100.00	69.05
20: BfFucH	21.91	24.25	25.14	25.14	26.21	22.44	27.59	25.20	21.41	29.69	26.86	25.81	24.34	27.52	27.49	28.61	28.78	32.26	69.05	100.00

By multiple sequence alignment, most α-fucosidases have sequence identity in the high teens to low 30's with any other given α-fucosidase.

Taking AlfC (class A) as an example, the class B α-fucosidases with which it is *most divergent* is BT3798, with which it shares 19.05% sequence identity, a value which the reviewer might suggest is "virtually nothing" and/or suggestive of different folds.

Crystal structures of both these enzymes exist, however, and superpositions show they are virtually identical in overall structure (AlfC-cyan, BT3798 [PDB 3GZA]-magenta). When aligned, they have a C_αRMSD of only 1.3 Å, as shown below.

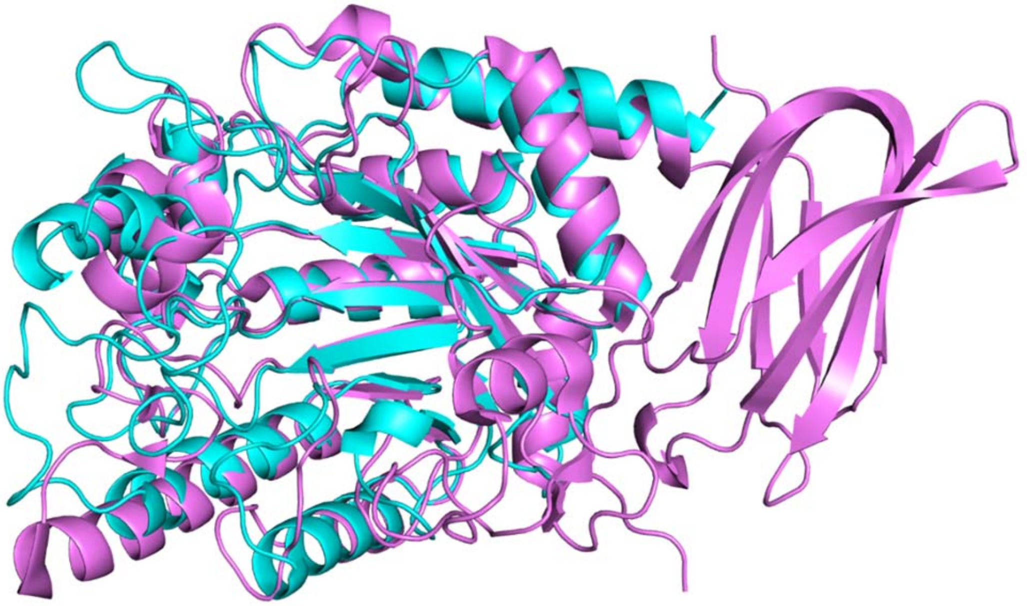

Furthermore, there is close alignment of the catalytic residues and many of the residues which bind fucose, as shown below.

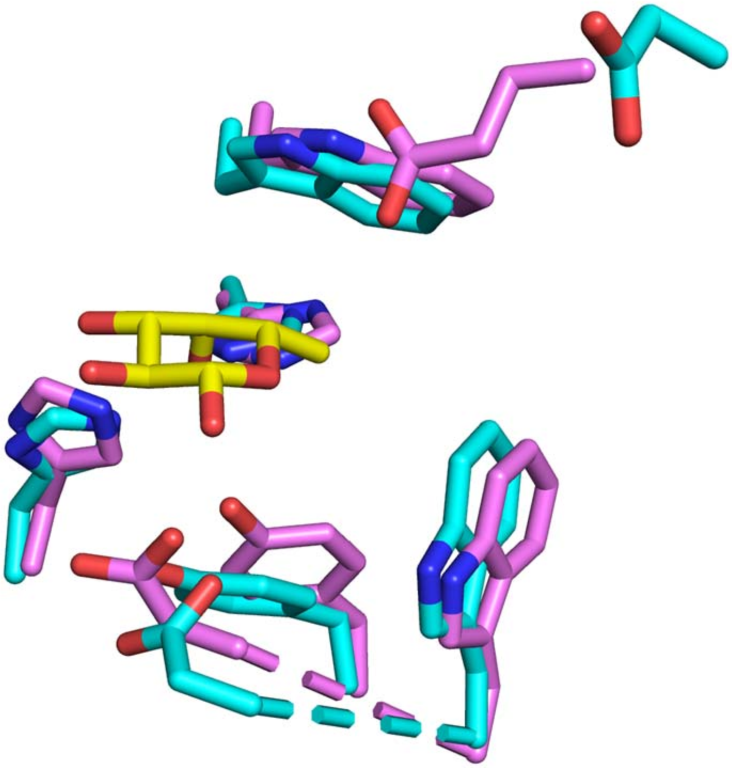

Of course, there are important differences in the "+1" site that binds the carbohydrate that fucose attaches to, likely responsible for the difference in substrate preference between these enzymes. However, the overall similarities in fold and catalytic residue positioning suggests that these enzymes bind their substrates differentially, then use a unified mechanism of hydrolysis, in a strong example of convergent evolution.

We feel that this subject is worthy of a future studies and a detailed review on the structure/function relationship of GH29 binding sites. However, we believe that this manuscript can be fully appreciated without delving further into the topic than currently present.

In fact, GH29-A are common in eukaryotes and GH29-B are generally of bacterial origin.

While it is true than GH29B are generally of bacterial origin, GH29A are well represented in all kingdoms, for example:

Eukaryotes: FucA1[15], BFK[16], FgFCO1[5], NcFuc[17]
Bacteria: AlfC, α-L-ftwt[18], Tmα-Fuc[19], BT2970[20]
Archaea: SsFuc[21]

As described above, where crystal structures exist, these enzymes appear to be subject to convergent evolution.

Notably, a broad specificity class B fucosidase from O. bacterium has been identified and characterized relatively recently, more details here Vainauskas et al, Scientific Reports 2018, 8.

In the paper this reviewer references[22], the authors never assign FucO to a GH29 class. Using the same method described above, phylogenetic analysis suggests this enzyme belongs to class A, not class B.

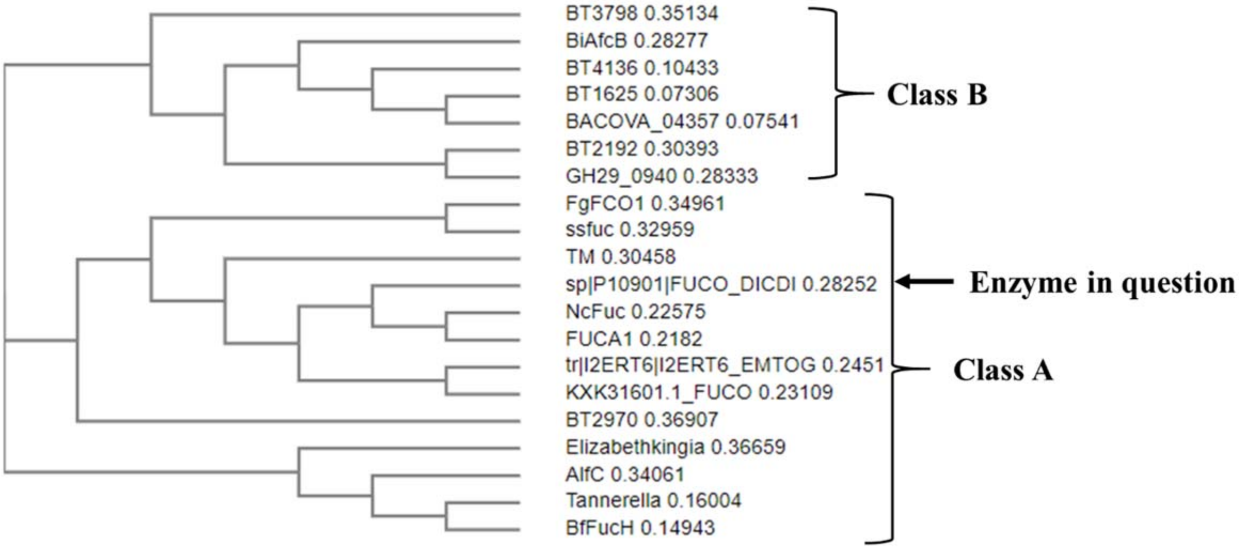

This is furthermore consistent with the observation that it hydrolyzes α(1-3,6)-linkages[22]. To our knowledge, class B enzymes have only been described to hydrolyze α(1-3,4)-linkages[6].

<u>Reviewer #3:</u>

Core-fucosylation play a significant role in glycobiology and in production of therapeutic antibodies. The manuscript from Wang, Sundberg and co-workers describe a solid study exploring the mechanism of the core fucose fucosidase AlfC and their mutants. The study also explore AlfC mutants that may act as a transfucosidase and additionally tolerate complex type N-glycans in contrast to the WT AlfC fucosidase. The authors investigate the mechanisms behind why the diffrent mutants can have different functions and substrate scope by deteremination of crystal structures, MD calculations, kinetic analysis, NMR and HDX-MS analysis. I would recommend publication after minor revision.

Figure S9: color assignment of WT vs mutant not the same in figure as in figure legend, please correct.

Thank you for noticing this. The color assignment has been corrected in the figure legend.

The authors make co-crystal structures with the Fuc-a-1,6-GlcNAc disaccharide, it would be

desirable to use the disaccharide attached to a peptide backbone for ligand co-crystalization to confirm that the ligand is correctly oriented with regard to steric clash and conclusions explaining why other Fuc linkage connections would not be suitable.

With regard to AlfC mutants acting as transglycosidases, it would have been convincing to additionaly include a core fucose complex type N-glycan as ligand in a co-crystal structure that would confirm that the oligosaccharide fit in the more open conformation of the enzyme.

We fully agree with your desire for more crystal structures, including:
(i) Structures with peptide attached
(ii) Structures with more complete glycans

Solving these structures is a priority in our lab, and we are exploring ways of obtaining these structures, including by cryo-EM. Unfortunately, it requires synthesis and purification of non-trivial substrates in high yield, making it a slow and difficult pursuit. We hope to solve such structures in the future and publish them in a subsequent study.

Regarding the transglycosidase activity, have other complex type N-glycans than biantennary been used as substrates for the fucose translycosylation?

High-mannose N-glycans and several biantennary complex type N-glycans in the context of peptides and proteins, including antibody, have been tested previously and have been shown to be substrates of the fucosidase mutant (ref. 12 of the revised manuscript)[1]. We have not tested other complex type N-glycans, but we agree that it will be interesting to test additional complex type N-glycans, such as the triantennary complex type N-glycans to understand the structural requirement. We plan to perform this study in the future and hope to report the results in due course.

The manuscript is rather long.

We appreciate this concern. At points we considered breaking it into two manuscripts: one on hydrolytic activity and one on transfucosylation. However, we felt it was a more complete and compelling story to include all the data in one place. We hope readers will agree.

<u>Reviewer #4:</u>

This paper provides a careful and characterization of the structural enzymology of an enzyme that is important for its role in cell adhesion and blockage thereof and post-translational modifications of potential bioactive molecules. The authors provide a credible explanation for the relative rates of hydrolysis versus transglycosylation and made mutant versions that further probe the mechanism of action and support the rationales provided. The MD and HD exchange data buttress the arguments about the role of more open or closed states. Altogether the paper represents an impressive array of experiments that are well integrated into a compelling story.

Thank you for your feedback—we're glad you found the story compelling!

Technical points to be addressed before any publication:

For solid validation of the bound carbohydrates:
1)The crystallographic "Table 1" must list the real space CC's or Z scores for the bound carbohydrate ligands.

Real space CC scores for bound ligands have been added to Table S2. They are 0.94, 0.89, and 0.89 for fucose, 4NP-fuc, and Fucα1,6GlcNAc, respectively.

2)Supplemental information should contain polder maps of the bound ligands.

Polder maps for bound ligands have been generated and added to Supplemental information, reproduced below:

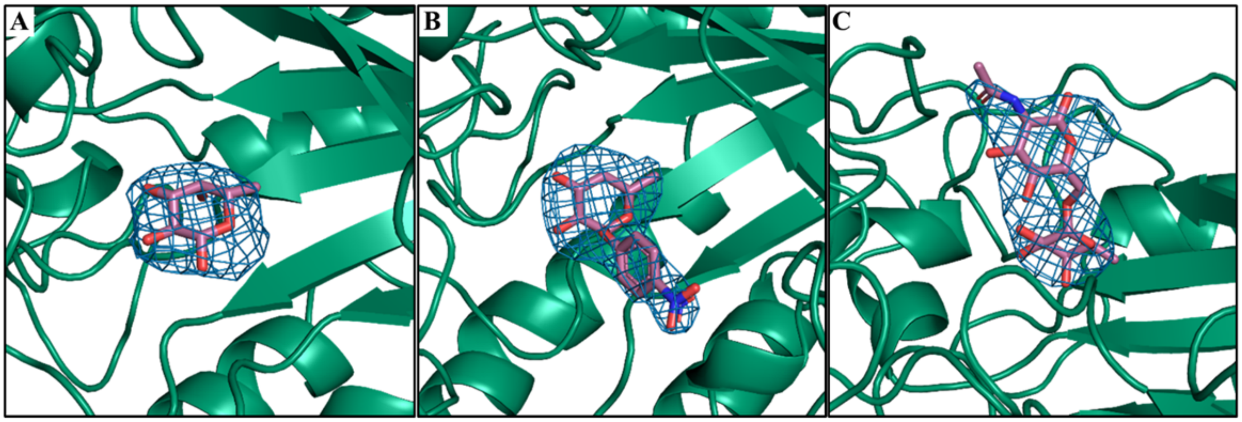

Figure S13. Polder maps were generated by omitting ligand atoms. **(A)** Fucose, σ = 5.5, carve = 2.3 Å; **(B)** 4NP-fuc, σ = 4, carve = 2.3 Å; **(C)** Fucα(1,6)GlcNAc, σ = 4, carve = 2.6 Å

3)The PDB entry ID's must be provided for the newly deposited works.

PDB entry ID's are present as the bottom line in Table S2. They have been additionally reproduced in the text under the "Data availability" section, reproduced below:

"The atomic coordinates have been deposited in the Protein Data Bank, www.pdb.org (PDB ID codes 6O18 [$AlfC_{WT}$], 6O1A [AlfC-fucose], 6O1C [AlfC-4NP-fuc], 6O1I [$AlfC_{E274A}$], 6O1J [$AlfC_{N243A}$-fucose], 6OHE [$AlfC_{D200A}$-Fucα1,6-GlcNAc])."

References

1 Li, C., Zhu, S., Ma, C. & Wang, L. X. Designer alpha1,6-Fucosidase Mutants Enable Direct Core Fucosylation of Intact N-Glycopeptides and N-Glycoproteins. *J Am Chem Soc* **139**, 15074-15087, doi:10.1021/jacs.7b07906 (2017).

2 Osanjo, G. *et al.* Directed evolution of the alpha-L-fucosidase from Thermotoga maritima into an alpha-L-transfucosidase. *Biochemistry* **46**, 1022-1033, doi:10.1021/bi061444w (2007).

3 Zeuner, B., Vuillemin, M., Holck, J., Muschiol, J. & Meyer, A. S. Loop engineering of an alpha-1,3/4-l-fucosidase for improved synthesis of human milk oligosaccharides. *Enzyme Microb Technol* **115**, 37-44, doi:10.1016/j.enzmictec.2018.04.008 (2018).
4 Sulzenbacher, G. *et al.* Crystal structure of Thermotoga maritima alpha-L-fucosidase. Insights into the catalytic mechanism and the molecular basis for fucosidosis. *J Biol Chem* **279**, 13119-13128, doi:10.1074/jbc.M313783200 (2004).
5 Cao, H., Walton, J. D., Brumm, P. & Phillips, G. N., Jr. Structure and substrate specificity of a eukaryotic fucosidase from Fusarium graminearum. *J Biol Chem* **289**, 25624-25638, doi:10.1074/jbc.M114.583286 (2014).
6 Sakurama, H. *et al.* Differences in the substrate specificities and active-site structures of two alpha-L-fucosidases (glycoside hydrolase family 29) from Bacteroides thetaiotaomicron. *Biosci Biotechnol Biochem* **76**, 1022-1024, doi:10.1271/bbb.111004 (2012).
7 Dawson, G. & Tsay, G. Substrate specificity of human alpha-L-fucosidase. *Arch Biochem Biophys* **184**, 12-23, doi:10.1016/0003-9861(77)90321-6 (1977).
8 DiCioccio, R. A., Barlow, J. J. & Matta, K. L. Substrate specificity and other properties of alpha-L-fucosidase from human serum. *J Biol Chem* **257**, 714-718 (1982).
9 O'Flaherty, R. *et al.* Aminoquinoline Fluorescent Labels Obstruct Efficient Removal of N-Glycan Core alpha(1-6) Fucose by Bovine Kidney alpha-l-Fucosidase (BKF). *J Proteome Res* **16**, 4237-4243, doi:10.1021/acs.jproteome.7b00580 (2017).
10 Trastoy, B. *et al.* Structural basis for the recognition of complex-type N-glycans by Endoglycosidase S. *Nat Commun* **9**, 1874, doi:10.1038/s41467-018-04300-x (2018).
11 Klontz, E. H. *et al.* Molecular Basis of Broad Spectrum N-Glycan Specificity and Processing of Therapeutic IgG Monoclonal Antibodies by Endoglycosidase S2. *ACS Cent Sci* **5**, 524-538, doi:10.1021/acscentsci.8b00917 (2019).
12 Ahmed, M. C., Papaleo, E. & Lindorff-Larsen, K. How well do force fields capture the strength of salt bridges in proteins? *PeerJ* **6**, e4967, doi:10.7717/peerj.4967 (2018).
13 Huang, J. *et al.* CHARMM36m: an improved force field for folded and intrinsically disordered proteins. *Nat Methods* **14**, 71-73, doi:10.1038/nmeth.4067 (2017).
14 Paschinger, K. & Wilson, I. B. H. Comparisons of N-glycans across invertebrate phyla. *Parasitology* **146**, 1733-1742, doi:10.1017/S0031182019000398 (2019).
15 Liu, S. W. *et al.* Identification of essential residues of human alpha-L-fucosidase and tests of its mechanism. *Biochemistry* **48**, 110-120, doi:10.1021/bi801529t (2009).
16 Hanfland, P. Characterization of B and H blood-group active glycosphingolopids from human B erythrocyte membranes. *Chem Phys Lipids* **15**, 105-124, doi:10.1016/0009-3084(75)90035-3 (1975).
17 Perrella, N. N., Withers, S. G. & Lopes, A. R. Identity and role of the non-conserved acid/base catalytic residue in the GH29 fucosidase from the spider Nephilingis cruentata. *Glycobiology* **28**, 925-932, doi:10.1093/glycob/cwy083 (2018).
18 Kovalova, T. *et al.* Active site complementation and hexameric arrangement in the GH family 29; a structure-function study of alpha-l-fucosidase isoenzyme 1 from Paenibacillus thiaminolyticus. *Glycobiology* **29**, 59-73, doi:10.1093/glycob/cwy078 (2019).
19 Tarling, C. A. *et al.* Identification of the catalytic nucleophile of the family 29 alpha-L-fucosidase from Thermotoga maritima through trapping of a covalent glycosyl-enzyme intermediate and mutagenesis. *J Biol Chem* **278**, 47394-47399, doi:10.1074/jbc.M306610200 (2003).
20 Lammerts van Bueren, A. *et al.* Analysis of the reaction coordinate of alpha-L-fucosidases: a combined structural and quantum mechanical approach. *J Am Chem Soc* **132**, 1804-1806, doi:10.1021/ja908908q (2010).

21 Cobucci-Ponzano, B., Mazzone, M., Rossi, M. & Moracci, M. Probing the catalytically essential residues of the alpha-L-fucosidase from the hyperthermophilic archaeon Sulfolobus solfataricus. *Biochemistry* **44**, 6331-6342, doi:10.1021/bi047495f (2005).
22 Vainauskas, S. *et al.* A novel broad specificity fucosidase capable of core alpha1-6 fucose release from N-glycans labeled with urea-linked fluorescent dyes. *Sci Rep* **8**, 9504, doi:10.1038/s41598-018-27797-0 (2018).

REVIEWERS' COMMENTS:

Reviewer #1 (Remarks to the Author):

I would like to thank authors for their responses to my question and clarification of some of my doubts. The manuscript was improved by addressing questions of all other reviewers. I find now the manuscript ready for publication.

Reviewer #2 (Remarks to the Author):

I thank the authors for a very thorough response to my queries.

In the matter of the differences between the 3D structure (or fold) of different fucosidases, I think there was a bit of a confusion in how my point was taken. The 3D structure, and I used 'fold' as a synonym for that, see "or fold", I was referring to is the tertiary structure, thus not the quaternary structure and not the binding site only, which indeed may share a high % identity and spatial orientation of the key residues, especially when the mechanism of hydrolysis is the same. My point was rather that the tertiary structures are different together with the proteins size. In this regard, I still stand behind my statement that a 20-30% range of sequence identity is far from a conserved overall structure, as it is considered as the minimum threshold to build a meaningful homology model. In this regard, so I do disagree with the statement,

"Pairwise sequence identity varies from 26.4% to 31.2%, which generally corresponds to a conserved overall structure."

Also, the above is in clear contradiction, in my opinion, with another statement in the authors' reply, namely that the homology model between BKF and 1ODU is somewhat uncertain in terms of validity, where in this specific case the overall sequence identity is 38%, with a steep increase within the active site region.

In terms of the structural alignment done with pyMol, also kindly represented in terms of 3D structure by the authors in their reply, the RMSD value of 1 Å seems quite low to me, especially in view of that specific image. This can be the result of the (automatic) elimination of the atoms that cause the RMSD value to fall out of the threshold set (by default) in pyMol. The authors may want to look at the number of atoms used to calculate the RMSD value and how it changes by setting the default to a higher threshold. That being said, the alignment shows high similarity in and around the active site, which, as I mentioned above, is expected in view of the same (or similar) hydrolysis mechanism and binding specificity of the whole glycan being digested.

Perhaps, instead, the reviewer means to suggest that different alpha fucosidases within the same family (GH29A) have different mechanisms of binding their substrates. This is almost certainly true as indicated by their different linkage specificities.

That is indeed what I meant, sorry if my statement was confusing. I am glad the authors agree with it and within this framework, I think it's an important point to acknowledge in the discussion of different types of fucosidases.

In regards to the length of the MD simulations I appreciate that the resources required to simulate those systems are substantial and may be not available to all researchers, yet that doesn't justify, in my opinion, that the interpretation or the weight of relatively short simulations in the results can or should be considered as from complete/sufficient sampling. Therefore, a note of warning should be added, indicating that the interpretation comes from limited sampling. This is especially important when single point mutations built on the wild type structure, as those may require further sampling to

reach conformational equilibration. Moreover, if conventional MD and enhanced sampling give different weights of open/closed populations, that is a clear sign that sampling in one (or both) simulations is incomplete, thus may be insufficient.

Also, the enhanced strength of salt-bridges is a common feature of many empirical (additive) force fields and not only of CHARMM. So, I still think that a note of caution should be also added.

In the matter of the MS data, I do appreciate the effort of producing figures that facilitate understanding by a broad audience, yet I do believe that the spectra in the figure in the main manuscript should be the ones coming out of the mass spectrometer, with an additional panel highlighting the region of interest and/or the overlaid lines image with a clear statement in the caption indicating that that panel is produced by retracing a graph for clarity. As a note, I have never doubted that the authors did the retracing with anything but the best intentions, yet these days is paramount that the original data are represented as they are, with no further manipulation, which, as also the authors underlined, may raise suspicions.

Reviewer #3 (Remarks to the Author):

I recommend publication of the revised manuscript by Wang, Sundberg and co-workers. It would be desirable that additional substrates are tested for fucose transglycosylation activity.

Reviewer #4 (Remarks to the Author):

My comments have been well addressed and I feel the paper is suitable for publication.

We would like to thank reviewer #2 for the additional helpful critiques of our work. Below, we provide point-by-point responses to this reviewer's comments.

New reviewer comments appear in plain text
Author responses to the initial review (as quoted by the reviewer) appear in italicized text
Author responses to new reviewer comments appear in bold italicized text

Reviewer #2 (Remarks to the Author):

I thank the authors for a very thorough response to my queries.

In the matter of the differences between the 3D structure (or fold) of different fucosidases, I think there was a bit of a confusion in how my point was taken. The 3D structure, and I used 'fold' as a synonym for that, see "or fold", I was referring to is the tertiary structure, thus not the quaternary structure and not the binding site only, which indeed may share a high % identity and spatial orientation of the key residues, especially when the mechanism of hydrolysis is the same. My point was rather that the tertiary structures are different together with the proteins size. In this regard, I still stand behind my statement that a 20-30% range of sequence identity is far from a conserved overall structure, as it is considered as the minimum threshold to build a meaningful homology model. In this regard, so I do disagree with the statement,

"Pairwise sequence identity varies from 26.4% to 31.2%, which generally corresponds to a conserved overall structure."

Also, the above is in clear contradiction, in my opinion, with another statement in the authors' reply, namely that the homology model between BKF and 1ODU is somewhat uncertain in terms of validity, where in this specific case the overall sequence identity is 38%, with a steep increase within the active site region.

We apologize for the confusion on our part of how we understood the reviewer's initial point on 3D structure/fold. We agree, in principle, with what the reviewer states above. This conversation between authors and reviewers is, however, limited to the reviews and not the manuscript proper. We find no statements in the manuscript that are in strict contradiction to what the reviewer states here and, therefore, have not made further changes to the manuscript in this regard.

In terms of the structural alignment done with pyMol, also kindly represented in terms of 3D structure by the authors in their reply, the RMSD value of 1 Å seems quite low to me, especially in view of that specific image. This can be the result of the (automatic) elimination of the atoms that cause the RMSD value to fall out of the threshold set (by default) in pyMol. The authors may want to look at the number of atoms used to calculate the RMSD value and how it changes by setting the default to a higher threshold. That being said, the alignment shows high similarity in and around the active site, which, as I mentioned above, is expected in view of the same (or similar) hydrolysis mechanism and binding specificity of the whole glycan being digested.

Perhaps, instead, the reviewer means to suggest that different alpha fucosidases within the same family (GH29A) have different mechanisms of binding their substrates. This is almost certainly true as indicated by their different linkage specificities.

That is indeed what I meant, sorry if my statement was confusing. I am glad the authors agree with it and within this framework, I think it's an important point to acknowledge in the discussion of different

types of fucosidases.

Again, we feel that we are in agreement with the reviewer. We have added a sentence that acknowledges that enzymes within the GH29A family have different mechanisms of binding their substrates: "[This is in part due to the diversity in substrate specificity of GH29A enzymes], where enzymes with different linkage specificities necessarily have different mechanisms for binding their substrates." Phrase in [] is from previous manuscript version.

In regards to the length of the MD simulations I appreciate that the resources required to simulate those systems are substantial and may be not available to all researchers, yet that doesn't justify, in my opinion, that the interpretation or the weight of relatively short simulations in the results can or should be considered as from complete/sufficient sampling. Therefore, a note of warning should be added, indicating that the interpretation comes from limited sampling. This is especially important when single point mutations built on the wild type structure, as those may require further sampling to reach conformational equilibration. Moreover, if conventional MD and enhanced sampling give different weights of open/closed populations, that is a clear sign that sampling in one (or both) simulations is incomplete, thus may be insufficient.

Also, the enhanced strength of salt-bridges is a common feature of many empirical (additive) force fields and not only of CHARMM. So, I still think that a note of caution should be also added.

Although unlikely, we agree that it is possible that our limited sampling, which was necessitated by computational expense, could be potentially misleading and have, accordingly, added the following text to the manuscript: "… although, the computational expense of our comprehensive approach to analyzing the many AlfC mutants by this approach necessitated limited sampling and further sampling may be required to reach conformational equilibrium in every case."

We have also added a note of caution regarding salt-bridges: "Although empirical force fields tend to overestimate the strength of salt-bridges, this interaction is nearly identical to the salt-bridge observed in the crystal structure of BT2970, where R262 recruits the acid/base E288 to the active site (Figure S2)."

In the matter of the MS data, I do appreciate the effort of producing figures that facilitate understanding by a broad audience, yet I do believe that the spectra in the figure in the main manuscript should be the ones coming out of the mass spectrometer, with an additional panel highlighting the region of interest and/or the overlaid lines image with a clear statement in the caption indicating that that panel is produced by retracing a graph for clarity. As a note, I have never doubted that the authors did the retracing with anything but the best intentions, yet these days is paramount that the original data are represented as they are, with no further manipulation, which, as also the authors underlined, may raise suspicions.

We appreciate that the reviewer would like to see the spectra as they appear on the mass spectrometer computer. We found that our attempts to produce a figure as suggested, though, resulted in no representation that would be legible as a figure in the main manuscript. However, we stress that the data which were replotted have not been manipulated in any way, and source data have been provided so that these figures can be re-created by any reader. Nonetheless, we have added a statement in the caption that clearly indicates that the graphs have been overlaid and re-traced for clarity. We also would like to point out that we have published MS data depicted in an identical manner in numerous peer-reviewed journals, including **Nature Communications** ***(e.g., Figure***

9e in Trastoy et al. (2018)9:1874) with no editor, reviewer or reader concerns. We find that depicting this MS data as such is the best way in which to represent the data to the reader.
